# From predation to function: how myxobacteria drive soil microbial community dynamics and ecological functions

**DOI:** 10.1128/aem.01922-25

**Published:** 2025-12-03

**Authors:** Wei Dai, Yang Liu, Zhongli Cui, Weishan Li, Hui Wang

**Affiliations:** 1 College of Tobacco Science and Engineering, Zhengzhou University of Light Industry 117776, Zhengzhou, China; 2 CAS Key Laboratory of Soil Environment and Pollution Remediation, Institute of Soil Science, Chinese Academy of Sciences 74586 , Nanjing, China; 3 Key Laboratory of Agricultural Environmental Microbiology of Ministry of Agriculture, Nanjing Agricultural University 70578, Nanjing, China; 4 Nanjing Institute of Environmental Sciences, Ministry of Ecology and Environment 159387, Nanjing, China; University of Delaware, Lewes, Delaware, USA

**Keywords:** predatory myxobacteria, soil microcosm, microbial community dynamics, ecological functions

## Abstract

Predatory myxobacteria play a crucial role as key predators in the soil microbial food web, influencing microbial community structure and functions. However, the mechanisms through which myxobacteria regulate these communities and their associated ecological functions remain inadequately understood. This study aims to investigate the regulatory effects of myxobacterial predation on soil bacterial composition, community dynamics, and ecological functions using a controlled soil microcosm system. The results show that the predatory myxobacterium *Corallococcus* sp. EGB exhibits strong predatory activity against common agricultural soil bacteria, particularly *Acinetobacter lwoffii* and *Bacillus subtilis*. In the microcosm systems, myxobacterial predation significantly altered bacterial community composition, diversity, and carbon metabolism (*P* < 0.05). In soils with high microbial abundance, myxobacterial predation reduced niche breadth (*P* < 0.05) and decreased the contribution of stochastic processes to community assembly. Prolonged incubation also increased extracellular enzyme activities and organic carbon mineralization rates (*P* < 0.05). Additionally, myxobacterial predation disrupted several metabolic pathways and modulated the functional distribution of bacterial communities. These findings highlight the critical role of myxobacteria in shaping soil microbial community dynamics and ecological functions, providing new insights for sustainable soil management and agricultural optimization.

**IMPORTANCE:**

Soil microbial communities drive nutrient cycling and carbon transformation, underpinning soil fertility and ecosystem function. Although microbial interactions are key regulators of soil processes, the ecological roles of predatory myxobacteria in modulating community composition and dynamics remain poorly understood. Here, we provide preliminary evidence that predation by *Corallococcus* sp. EGB reshapes bacterial community composition, alters functional potential, and influences carbon cycling, particularly in soils with high microbial abundance. By linking microbial predation to community dynamics and soil biogeochemical processes, this study advances understanding of the ecological significance of predatory myxobacteria and underscores their potential role in sustainable soil management.

## INTRODUCTION

Soil is one of the most complex and functionally diverse ecosystems on Earth, playing a critical role in global climate regulation, plant productivity, and ecological balance (1). Soil microbial communities, essential to soil health and ecosystem functions, are involved in key processes, such as nutrient cycling, carbon sequestration, and soil structure formation (2). Within these communities, predatory microorganisms, particularly myxobacteria, have attracted increasing attention (3, 4). The interactions between predators and prey in the soil microbial food web have profound effects, regulating the composition and functions of microbial communities, which, in turn, influence soil ecological processes. For instance, soil protozoa promote detritus decomposition by preying on bacteria and fungi, contributing to the carbon cycle (5, 6). Similarly, increased abundance of T4-like phages can cause bacterial mortality and suppress soil organic carbon mineralization (7), while nematode predation and competition affect microbial phosphorus dynamics (8). Despite the well-documented role of predatory myxobacteria in nutrient cycling, their broader ecological impacts on microbial community dynamics and soil functions remain underexplored (9).

Myxobacteria are bacterial predators that prey upon diverse microorganisms, including bacteria and fungi, primarily through the secretion of enzymes and antibiotics (10–12). Predation is the principal ecological strategy by which myxobacteria modulate microbial community composition and functional dynamics, whereas aggregation primarily reflects a developmental response to starvation. Through predation, myxobacteria not only directly reduce prey populations but also influence microbial diversity and metabolic functions, thereby shaping soil ecological processes. While previous studies have primarily focused on the immediate effects of predation on microbial communities (13–15), the broader ecological roles of myxobacteria—including their impacts on soil carbon metabolism, extracellular enzyme activity (EEA), and nutrient cycling—are still poorly understood, particularly regarding their contributions to the long-term stability and resilience of soil ecosystems.

Ecological theory suggests that the role of predators in food webs becomes more pronounced as productivity increases (16, 17). Variations in the diversity and abundance of soil microbial communities can influence predator behavior and the efficiency of energy flow (18); consequently, myxobacteria are likely to act as important regulators of soil ecological functions. Indeed, correlations between myxobacterial diversity and bacterial community diversity have been observed across diverse systems (19–21), but the mechanistic pathways linking predation to ecological function are not fully resolved.

This study aims to evaluate the ecological consequences of myxobacterial predation in soil ecosystems by integrating microbial community profiling and soil functional analyses. Accordingly, we propose two hypotheses: (H1) Myxobacterial predation reshapes bacterial community structure and diversity by suppressing specific prey populations; (H2) Such predation indirectly modulates soil ecological functions, particularly carbon cycling and nutrient turnover, through changes in community characteristics. To test these hypotheses, we combined metagenomic sequencing, microbial community assembly, EEA assays, and carbon metabolism analysis. This study seeks to investigate how myxobacterial predation influences the structure, diversity, and functional potential of soil microbial communities, thereby enhancing the understanding of the ecological role of myxobacteria and providing theoretical insights for sustainable soil management.

## MATERIALS AND METHODS

### Soil sampling collection and isolation of potential prey bacteria

Soil samples were collected from four typical agricultural regions in China, with three independent sampling points per region (a total of 12 samples). The basic properties of the soils at each sampling site are presented in Table 1. A cylindrical soil sampler (5 cm in diameter) was used to collect 10 kg of surface soil (0–20 cm depth) at each sampling point. The samples were immediately transported to the laboratory on dry ice and sieved (<2 mm) to remove plant debris and other contaminants. All samples were stored at 4°C until further analysis.

**TABLE 1:** Description of the basic condition of the soil tested

Parameter	Information for field site			
	Changshu (CS)	Fengqu (FQ)	Hailun (HL)	Yingtan (YT)
Longitude	123.63	113.16	126.91	116.91
Latitude	31.55	35.06	47.45	28.25
MAT (°C)	15.5	13.9	1.5	17.6
MAP (mm)	1038	605	550	1795
Climate type	Subtropical monsoon climate	Temperate monsoon climate	Temperate monsoon climate	Subtropical monsoon climate
Soil type	Paddy soil	Chao soil	Black soil	Red soil
pH	7.03	8.69	6.09	5.35
Ec (μS/cm)	168	113	32.55	25.8
SOC (g/kg)	23.49	7.14	23.45	11.85
TN (g/kg)	2.13	0.81	1.96	1.23
TP (g/kg)	0.96	1.23	0.86	0.66
TK (g/kg)	19.57	19.05	21.41	14.91
AP (mg/kg)	28.47	29.54	30.43	13.01
AK (mg/kg)	147.45	130.91	194.41	81.04
NO_3_^-^-N (mg/kg)	22.45	16.27	1.48	3.25
NH_4_^+^-N (mg/kg)	13.22	0.959	15.45	12.09
Ca^2+^ (cmol/kg)	14.98	34.46	17.58	4.47
Mg^2+^ (cmol/kg)	2.88	2.61	4.48	1.23
K^+^ (cmol/kg)	0.38	0.34	0.51	0.21
Na^+^ (cmol/kg)	0.57	0.18	0.21	0.22

Subsequently, 5 g of each soil sample was placed into sterile conical flasks containing 45 mL of sterile water and glass beads to prepare soil suspensions. The suspensions were incubated at 180 rpm and 30°C for 2 hours. After incubation, the suspensions were diluted to concentrations of 10^–3^, 10^–4^, 10^–5^, and 10^–6^ using the gradient dilution plating method (13). For each dilution, 0.1 mL of the suspension was spread onto lysogeny broth (LB), nutrient agar (NA), and yeast extract mannitol agar (YMA) solid media, with five replicate plates per concentration. All plates were incubated at 28°C until colonies appeared.

### Assessment of predatory activity of myxobacteria on soil bacteria

The predatory activity of myxobacteria on agricultural soil bacteria was evaluated using the lawn predation method (15). The myxobacterium strain *Corallococcus* sp. EGB, provided by Professor Zhongli Cui from Nanjing Agricultural University, and 48 potential prey bacterial strains isolated from typical agricultural soils were used in this study.

Each bacterial strain was cultured independently in LB and YMA liquid media for 24–48 hours. After incubation, cultures were centrifuged at 4,000 rpm for 30 minutes to collect cell pellets, which were washed twice with TM liquid medium (50 mmol/L Tris, pH 7.8; 10 mmol/L MgSO_4_) and resuspended prior to inoculation. To standardize biomass across strains, cell suspensions were adjusted to an OD_600_ of 1.0–1.2. Prey suspensions were spread on 14 cm diameter WAX agar plates (CaCl_2_·2H_2_O 1 g, HEPES 0.5 g, agar 15 g, water 1,000 mL, pH 7.2) to form uniform bacterial lawns, with three replicates per strain.

*Corallococcus* sp. EGB was grown in CTT liquid medium (Casein Peptone 10 g, MgSO_4_·7H_2_O 1.97 g, KH_2_PO_4_/K_2_HPO_4_ Buffer [pH 7.6] 1 mmol/L, Tris·HCl Buffer [pH 7.6] 10 mmol/L, H_2_O 1,000 mL, pH 7.6) at 30°C for 5–7 days until high-density cultures (OD_600_ ≈ 2) were obtained, with three replicates per treatment. Cells were harvested by centrifugation at 4,000 rpm for 30 minutes, washed with TPM medium (50 mmol/L Tris, pH 7.8, 1 mmol/L MgSO_4_, 1 mmol/L potassium phosphate), and resuspended in TPM medium. Ten microliters (10 µL) of myxobacterial suspension was spotted onto each prey lawn, with five replicates per treatment. Predation diameter was measured every 24 hours until 120 hours, with the change in predation diameter serving as an indicator of predatory activity.

### Construction of soil bacterial community microcosm

To construct the soil bacterial community microcosm, 30 soil samples (approximately 50 g each) were collected, wind-dried, and sieved (< 2 mm), then sterilized by autoclaving (121°C for 20 min). Sterilization effectiveness was validated through culture-based methods. Soil moisture was adjusted to 60% of the field capacity, and samples were pre-incubated in the dark at 25°C for 3 days to stabilize colonization.

A defined consortium of living bacterial strains (prey community) was then inoculated into the sterilized soils. For predation treatments, 0.2 mL of *Corallococcus* sp. EGB suspension was added, while control treatments received 0.2 mL of sterile water. Each treatment was replicated three times. Destructive sampling was performed at various time points post-inoculation, with samples stored at 4°C for DNA extraction.

To examine the effect of myxobacterial predation under varying microbial abundances, 60 additional sterilized soil samples were inoculated with bacterial suspensions representing high, medium, and low microbial abundances (five replicates per treatment). After 48 hours of incubation at 30°C, soil samples were collected and CO_2_ concentrations were measured using a gas analyzer. Soil DNA was extracted for microbial community analysis. Sample labels ending in 2, 5, 9, and 15 correspond to incubation periods of 2, 5, 9, and 15 days following treatment, respectively. The supplemental methods provide more details.

### Analysis of carbon metabolic function and EEA in soil microbial communities

For carbon metabolic function analysis (22), 10 g of fresh soil per treatment (three replicates per treatment) was mixed with 100 mL of 0.05 M phosphate buffer and shaken for 30 minutes to fully suspend the soil. Then, 1 mL of the suspension was diluted to 10^−3^, and this diluted solution was added to each well of a BIOLOG ECO plate. The plate was incubated for 24–168 hours, and absorbance at 590 nm was measured. The average well color development (AWCD) was calculated, and the time curve of carbon source utilization was plotted.

The EEA in the soil was measured using a microplate fluorescence assay (23). First, 100 mL of homogeneous soil suspension (three replicates per treatment) was mixed with 50 mL substrate solution and 50 mL buffer solution, added to a 96-well plate. The plate was incubated at 28°C for 30 minutes. After incubation, fluorescence intensity was measured using a multifunctional enzyme reader with an excitation wavelength of 360 nm and an emission wavelength of 450 nm. The substrate conversion rate was calculated per hour per gram of sample, with units of nmol MUF/g/h or nmol AMC/g/h. EEAs included β-glucosidase (BG), cellobiohydrolase (CBH), and β-xylosidase (BX) related to the carbon cycle; leucine aminopeptidase (LAP), N-acetyl-β-D-galactosaminidase (NAGA), and N-acetyl-β-D-glucosaminidase (NAG) related to the nitrogen cycle; and alkaline phosphatase (AP) related to the phosphorus cycle. The EEA data were calculated in a standardized form to reflect the relative activity of different enzymes. The supplemental methods provide more details.

### High-throughput sequencing and data analysis

This study employed high-throughput sequencing technology to analyze soil microbial communities. Initially, 16S rRNA amplicon absolute quantification sequencing was used to analyze the microbial communities in synthetic bacterial microcosms (24, 25). Standardized insertion sequences were introduced into the sample DNA to enable absolute quantification. The V4-V5 regions and insertion sequences were sequenced using the Illumina HiSeq platform. Data processing included quality trimming, sequence merging, operational taxonomic unit (OTU) classification, and annotation using the Ribosomal Database Project database. To assess bacterial community similarity, Bray-Curtis dissimilarity metrics and Principal Coordinate Analysis (PCoA) were applied. Additionally, the FAPROTAX tool was used to predict microbial functional profiles based on taxonomic composition (26).

For soil samples with varying microbial abundance gradients, 16S rRNA amplicon sequencing was conducted. DNA was extracted using the FastDNA Soil Spin Kit, and the V4-V5 region of the 16S rRNA gene was PCR amplified and sequenced on the Illumina MiSeq PE300 platform. Sequencing data were quality filtered, and chimeric sequences were removed using Uchime (27). OTU classification was performed with the QIIME software package (28), and taxonomic annotation was carried out using the SILVA reference database.

For metagenomic analysis, DNA was extracted from high-abundance soil samples and sequenced on the Illumina NovaSeq platform with paired-end 150 bp sequencing (PE150). Raw sequencing data were quality filtered and classified, with functional annotation performed using Kraken2 (29) and mmseqs2 (30), and gene function predicted with MetaGeneMark (31). Functional gene annotation was carried out using KEGG, CAZy, MCyc, NCyc, PCyc, and SCyc databases, with a focus on genes associated with carbon, nitrogen, phosphorus, and sulfur cycles. Further details are provided in the supplemental methods.

### Statistical analysis

For microbial community analysis, alpha diversity indices (OTU richness and Shannon index) were calculated for each treatment group (three biological replicates per treatment) using R software. Bray–Curtis distance matrices were constructed based on the absolute and relative abundance of OTUs, and PCoA was used to visualize community structure. Group differences were assessed using PERMANOVA (ADONIS). Although formal tests for homogeneity of group dispersions (e.g., betadisper) were not performed, visual inspection suggested that dispersion was generally comparable across treatments.

For metagenomic functional analyses, functional gene abundances were compared across treatments using t-tests or one-way ANOVA, assuming normality and homogeneity of variance. Post hoc comparisons following ANOVA were performed using Tukey’s HSD to control the family-wise error rate.

The effects of predatory bacteria and microbial abundance on soil EEA were evaluated using two-way ANOVA, with treatment and time as factors. Normality and homogeneity of variance were checked prior to analysis. To evaluate the effects of predatory bacteria and microbial abundance on soil EEA, two-way ANOVA with treatment and time as factors was applied, with normality and homogeneity of variance checked. CO_2_ efflux measurements were analyzed separately at each time point using one-way ANOVA, followed by Tukey’s HSD for post hoc comparisons. Differences in relative abundances of soil bacterial communities across microbial abundance gradients were analyzed using ANOVA, while β-diversity was evaluated based on Bray–Curtis distance metrics.

The βNTI index was used to evaluate bacterial community assembly processes between treatments. A βNTI value of |βNTI| > 2 indicates a deterministic process, and |βNTI| < 2 indicates a random process. The Raup–Crick matrix (RCbray) was used to analyze random processes, and neutral model analysis was performed using the “vegan” R package. Levin’s niche width (B) index was calculated, with 1,000 permutations for evaluation.

## RESULTS

### Predation of typical farmland soil bacteria by myxobacteria

**Fig 1:**
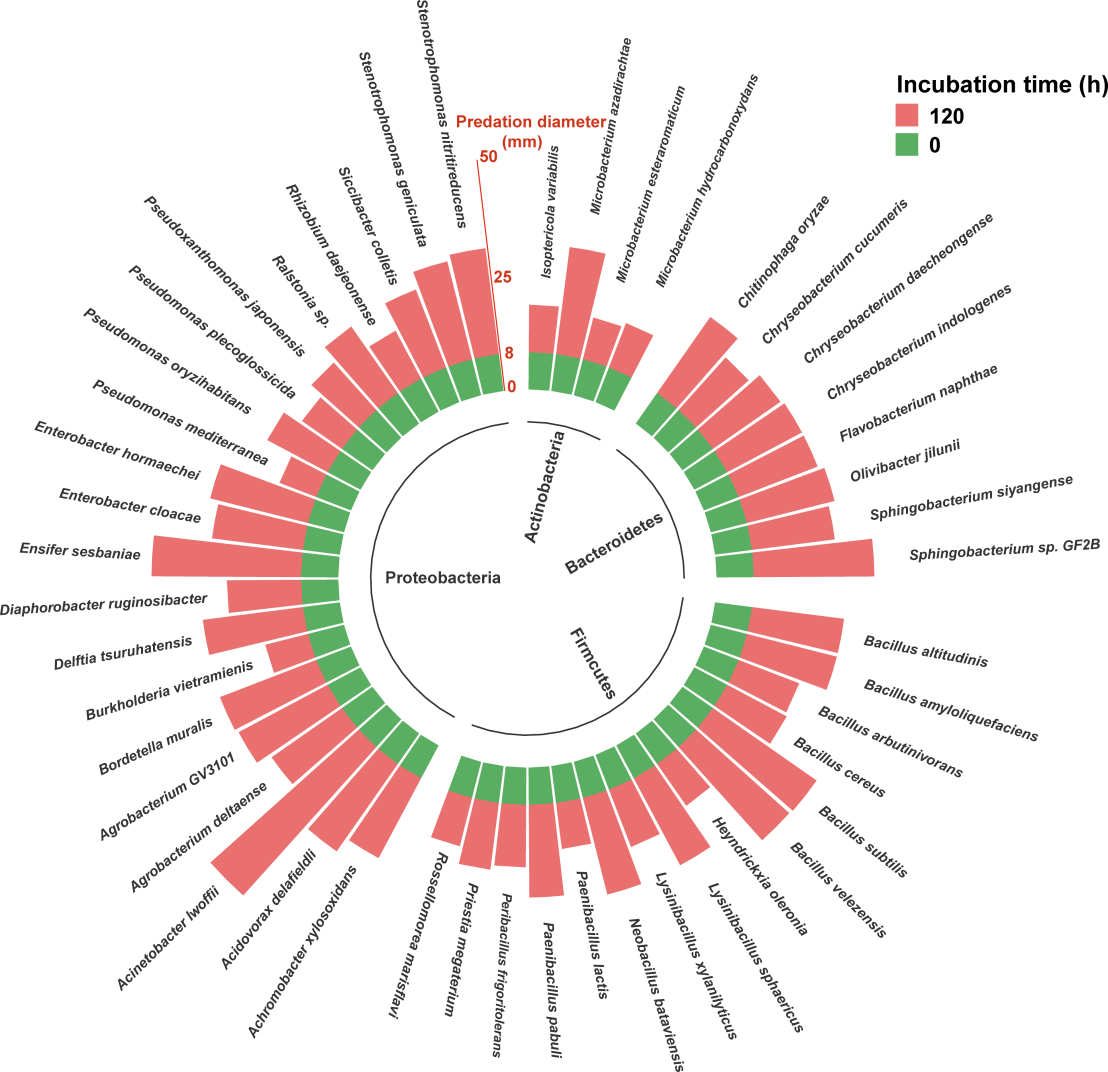
The predatory activity of myxobacteria (*Corallococcus* sp. EGB) on 48 typical soil bacteria from agricultural fields was assessed. The bar chart illustrates the predation diameters of myxobacteria on different strains, with green representing the initial predation diameter and red indicating the predation diameter for each strain after 120 hours.

A total of 48 bacterial strains were isolated from agricultural soil samples (Fig. 1) and identified through 16S rRNA sequencing. These strains were classified into four phyla: Proteobacteria, Actinobacteria, Firmicutes, and Bacteroidetes, representing 31 genera. The most abundant genera identified include *Bacillus*, *Microbacterium*, *Paenibacillus*, and *Pseudomonas*, which are key components of the soil microbial community. Predation assays revealed varying levels of susceptibility among these bacterial strains to *Corallococcus* sp. EGB. Here, “strong” versus “weak” predation was defined based on the diameter of lysis zones in the lawn predation assays, measured every 24 hours. Strong predation (≥23 mm) was observed against *Acinetobacter lwoffii*, *Ensifer sesbaniae*, *Bacillus subtilis*, *Microbacterium azadirachtae*, and *Stenotrophomonas nitritireducens*, while weaker predation (≤10 mm) occurred against *Microbacterium esteraromaticum*, *Burkholderia vietramienis*, and *Pseudomonas mediterranea*. Notably, significant differences in predation were observed even within the *Microbacterium* genus, with *Microbacterium azadirachtae* being more susceptible to *Corallococcus* sp. EGB than *Microbacterium esteraromaticum*.

### Effects of myxobacterial predation on soil bacterial community composition and diversity

**Fig 2:**
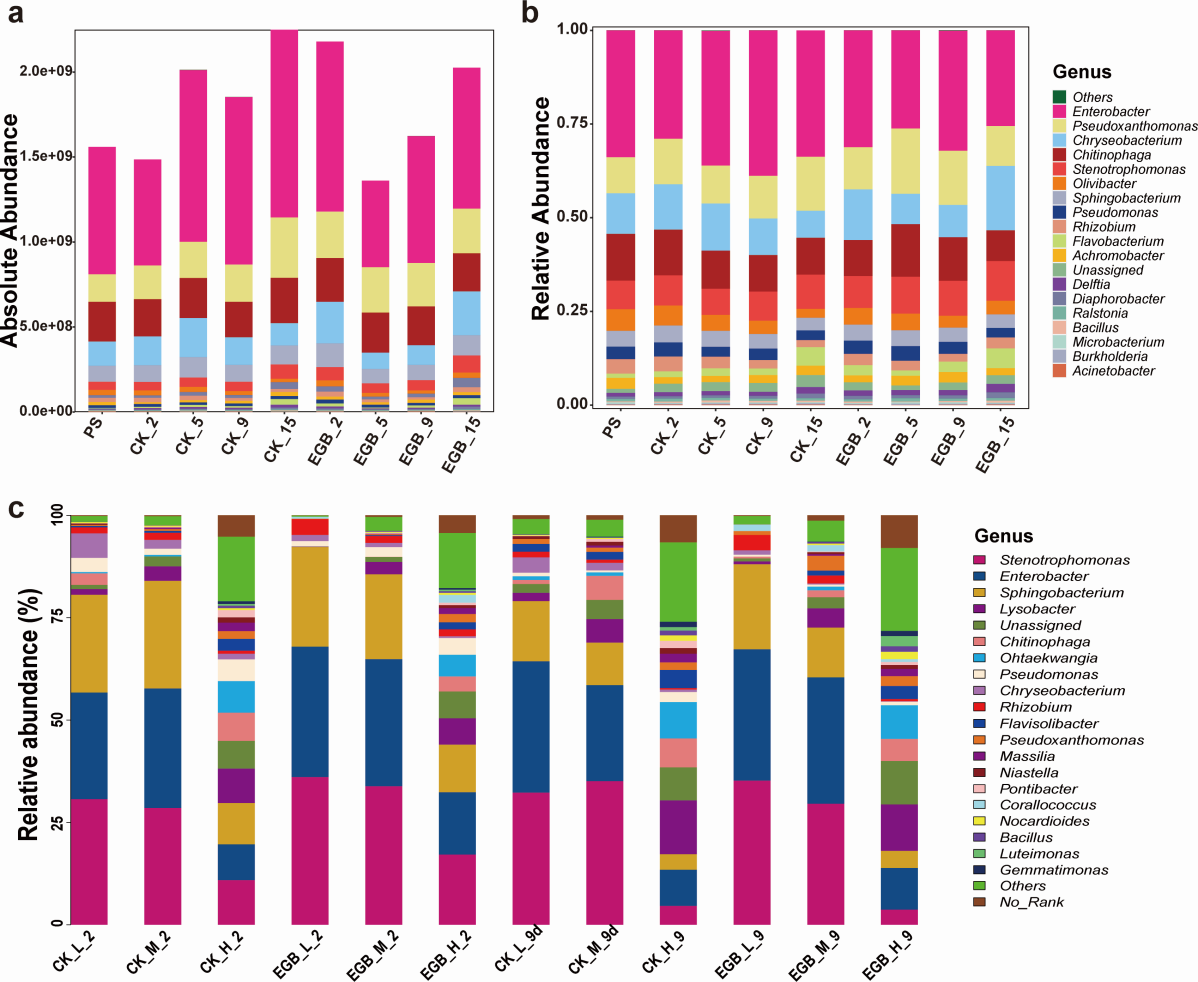
Bacterial community composition in the soil microcosm system. The synthetic bacterial communities in the soil microcosm are presented based on absolute abundance (**a**) and relative abundance (**b**). The bacterial community composition in the soil microcosm across different microbial abundance gradients (**c**). PS: primary sample, CK: control treatment, EGB: myxobacteria treatment; L: low microbial abundance, M: medium microbial abundance, H: high microbial abundance; the number represents incubation time.

In synthetic bacterial community microcosms, all 20 added genera were detected. The dominant genera, based on both absolute and relative quantification, included *Enterobacter* (46.23% in absolute, 31.78% in relative abundance), *Pseudoxanthomonas* (13.47%/12.37%), *Chitinophaga* (12.92%/10.86%), *Chryseobacterium* (9.43%/11.09%), and *Stenotrophomonas* (3.58%/8.64%) (Fig. 2). Two-way ANOVA indicated that both myxobacterial predation and incubation time significantly affected the abundances of *Enterobacter*, *Delftia*, and *Stenotrophomonas*, while *Flavobacterium* and *Olivibacter* were only influenced by incubation time (Table S2). Notably, the absolute abundance of *Corallococcus* sp. EGB (*Corallococcus*) was negatively correlated with most soil bacteria, except for *Microbacterium* and *Achromobacter* (Fig. S1), suggesting a potential predatory effect of *Corallococcus* sp. EGB on other soil bacteria.

**Fig 3:**
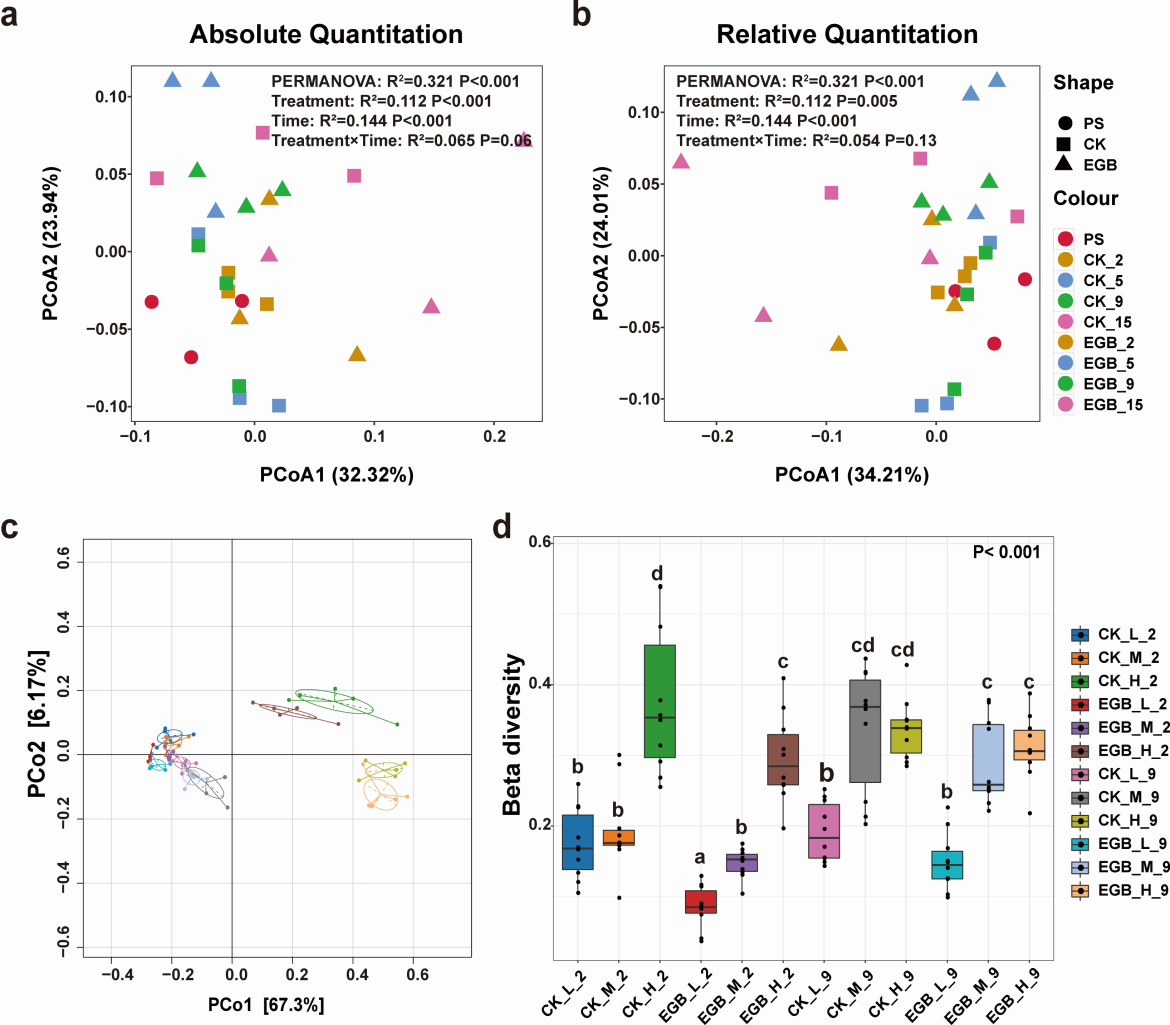
Bacterial community structure in the soil microcosm system. PCoA of the synthetic bacterial communities in the soil microcosm based on absolute abundance (**a**) and relative abundance (**b**). Bacterial community structure (**c**) and β diversity analysis (**d**) across different microbial abundance gradients in the soil microcosm. PS: primary sample, CK: control treatment, EGB: myxobacteria treatment; L: low microbial abundance, M: medium microbial abundance, H: high microbial abundance; the number represents incubation time. Statistical significance was assessed using PERMANOVA. The effects of treatment, time, and their interaction were evaluated by two-way PERMANOVA, with R^2^ and *P* values shown in the figure.

The α-diversity analysis showed no significant difference in diversity between absolute and relative abundances (t-test, *P* > 0.05) (Fig. S2). While the richness index (Richness) remained unchanged across treatments (*P* > 0.05), the Shannon diversity index was significantly higher with myxobacterial predation after 15 days of incubation (*P* < 0.05). PCoA further revealed significant differences in bacterial community structure between treatments (*P* < 0.001), with both myxobacterial predation and incubation time significantly influencing community composition (*P* < 0.05) (Fig. 3).

In soil microcosms with varying microbial abundance gradients, *Proteobacteria* (67.88%), *Bacteroidetes* (28.88%), and *Actinobacteria* (1.82%) dominated (Fig. S3). Myxobacterial predation increased *Proteobacteria* abundance while reducing *Bacteroidetes*. At the genus level, *Stenotrophomonas* (24.81%), *Enterobacter* (23.3%), *Sphingobacterium* (15.24%), *Lysobacter* (5.03%), and *Chitinophaga* (2.91%) were most abundant (Fig. 2c). Predation boosted *Enterobacter* and *Rhizobium* while decreasing *Chryseobacterium*. Significant differences in community richness and diversity were observed (*P* < 0.0001), with higher values in high microbial abundance soils (Fig. S2c and d). Predation reduced richness and diversity in high abundance soils, particularly after 2 days (*P* < 0.05). ADONIS analysis (Fig. 3d) revealed significant β-diversity differences (*P* < 0.001), with predation reducing community variability, especially in high abundance soils after 2 days (*P* < 0.05).

### Effects of myxobacterial predation on carbon metabolism of soil microbial communities

**Fig 4:**
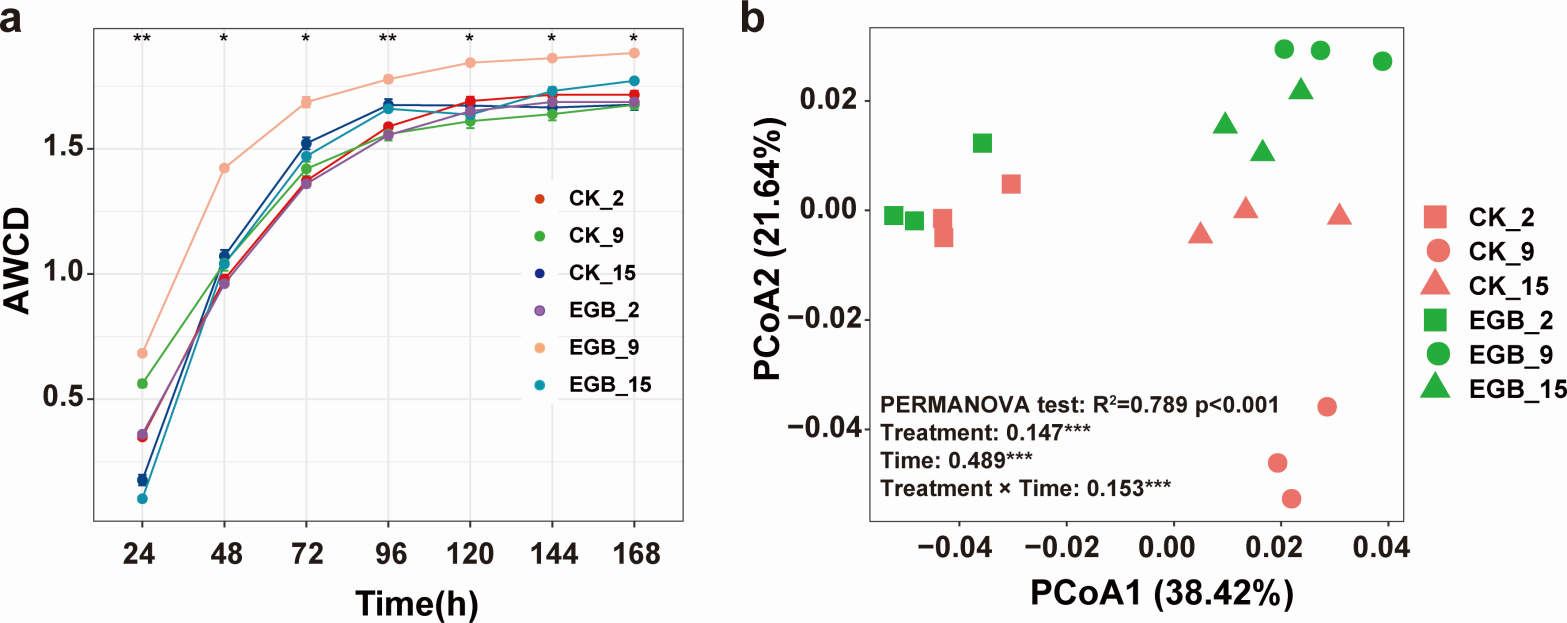
Changes in the synthetic bacterial community AWCD in the soil microcosm over incubation time (**a**) and its carbon metabolic functional structure (**b**). CK: control treatment, EGB: myxobacteria treatment; the number represents incubation time. Statistical significance was assessed using PERMANOVA. The effects of treatment, time, and their interaction were evaluated by two-way PERMANOVA, with R^2^ and *P* values shown in the figure; asterisks represent significance (*P < 0.05; **P < 0.01;***P < 0.001).

Myxobacterial predation significantly affected the carbon metabolism functions of synthetic bacterial communities in soil microcosms (Fig. 4). The AWCD values for EGB_9 (1.88 ± 0.01) and EGB_15 (1.77 ± 0.02) were higher than those for the control groups CK_9 (1.68 ± 0.03) and CK_15 (1.68 ± 0.37), indicating enhanced overall carbon utilization. PCoA of 168-hour absorbance data revealed significant differences in carbon metabolism across treatments (*P* < 0.001), with myxobacterial predation, incubation time, and their interactions all influencing carbon utilization (*P* < 0.05). Further analysis of carbon source contributions to the PCoA axes (Table S3) identified glucose-1-phosphate and β-methyl-D-glucoside as major contributors to the observed metabolic shifts, indicating that myxobacterial predation alters the potential for phosphorylated sugar and glycoside utilization within the microbial community.

### Effects of myxobacterial predation on the assembly process of soil bacterial communities

**Fig 5:**
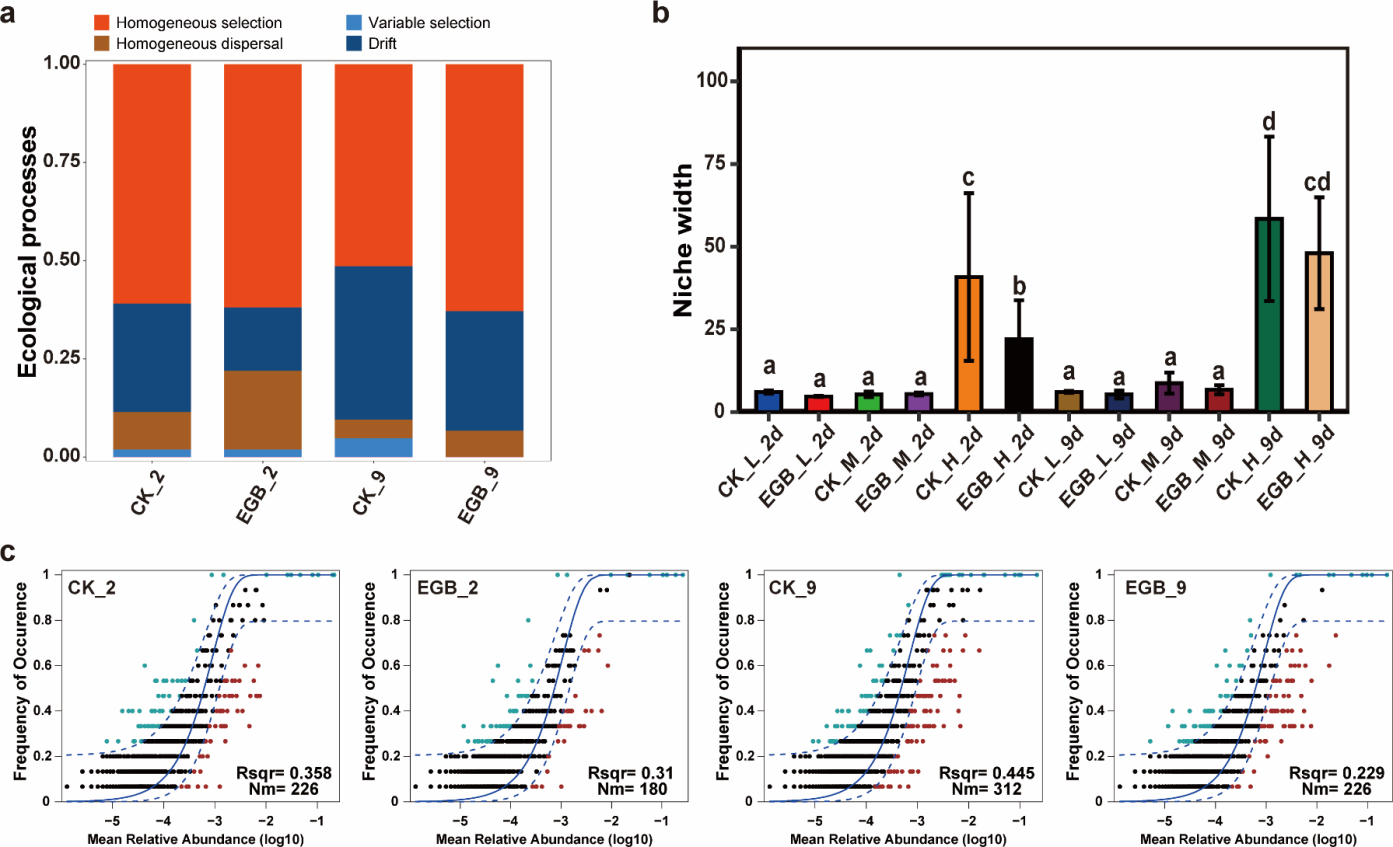
Assembly processes of bacterial communities in soil microcosms across different microbial abundance gradients. Assembly processes (**a**), niche width (**b**), and neutral model fitting (**c**) of soil bacterial communities under different treatments. CK: control treatment, EGB: myxobacteria treatment; L: low microbial abundance, M: medium microbial abundance, H: high microbial abundance; the number represents incubation time.

In soil microcosms with different microbial abundance gradients, bacterial community assembly was primarily driven by deterministic processes (Fig. 5). Homogeneous selection (51.42%–62.85%) was the dominant process, followed by drift (16.19%–39.04%) and homogeneous dispersal (4.76%–20%). After 2 days of incubation, myxobacterial predation did not significantly alter the balance between deterministic and stochastic processes but did affect the proportions of homogeneous dispersal and drift. After 9 days, predation increased deterministic processes by 6.67%, with a 10.47% increase in homogeneous selection. Neutral model analysis showed that myxobacterial predation reduced the influence of stochastic processes on community assembly. Specifically, after 2 days, the neutral model fit was higher for the control (R²=0.358) than for the myxobacterial treatment (R²=0.31). After 9 days, the fit remained higher for the control (R²=0.445) compared to the myxobacterial treatment (R²=0.229). Additionally, myxobacterial predation reduced the ecological niche width, especially in soils with high microbial abundance (*P* < 0.05).

### Effects of myxobacterial predation on EEA and organic carbon mineralization in soil

Myxobacterial predation significantly affected the EEA of soil microorganisms, particularly enzymes involved in carbon, nitrogen, and phosphorus cycling (Table 2). Two-way ANOVA showed that myxobacterial treatment significantly increased total EEA and nitrogen cycle-related enzyme activities (NE) (*P* < 0.05), while the microbial abundance gradient had no significant effect (*P* > 0.05). Myxobacterial predation notably altered the activities of BG, CBH, BX, NAGA, NAG, and AP (*P* < 0.05). The microbial abundance gradient significantly influenced CBH and BX activities (*P* < 0.05). Specifically, myxobacterial treatment increased EEA, NE, BX, and NAGA activities in soils with medium to high microbial abundance (*P* < 0.05), while promoting BG, CBH, and NAG activities. In soils with low microbial abundance, predation reduced AP activity (*P* < 0.05) but had no significant impact on the other enzymes.

**TABLE 2:** EEA in soil microuniverse with different microbial abundance gradients^*a*^

	CK_L_9	CK_M_9	CK_H_9	EGB_L_9	EGB_M_9	EGB_H_9	Myxobacteria (M)	Microbial abundance (MA)	M × MA
BG	44.20 ± 6.29a	33.37 ± 10.59a	37.29 ± 13.84a	43.48 ± 15.03a	56.66 ± 13.96a	54.70 ± 14.69a	*	ns	ns
CBH	12.63 ± 1.57ab	12.05 ± 3.01ab	5.9 ± 4.07a	26.92 ± 9.58c	19.51 ± 1.23bc	11.95 ± 3.01ab	**	**	ns
BX	9.77 ± 3.21ab	13.51 ± 7.16b	4.12 ± 2.27a	15.22 ± 1.78b	31.09 ± 4.20c	28.33 ± 5.44c	***	*	ns
LAP	93.78 ± 16.49b	66.90 ± 25.39ab	64.65 ± 16.97ab	39.68 ± 11.63a	63.32 ± 22.86ab	68.63 ± 6.47ab	ns	ns	*
NAGA	36.99 ± 8.46abc	13.20 ± 3.86a	21.88 ± 17.36ab	30.09 ± 9.61abc	50.61 ± 27.18c	44.70 ± 4.79bc	*	ns	ns
NAG	90.05 ± 35.08ab	17.05 ± 4.11a	60.53 ± 43.85ab	54.20 ± 34.01ab	103.01 ± 71.55b	132.55 ± 24.77b	*	ns	ns
AP	430.48 ± 130.88c	213.27 ± 30.75ab	280.82 ± 126.35bc	84.35 ± 40.88a	207.55 ± 126.76ab	300.28 ± 16.96bc	*	ns	**
EEA	2.27 ± 0.53ab	1.36 ± 0.36a	1.44 ± 0.68a	1.75 ± 0.44ab	2.52 ± 0.74c	2.49 ± 0.34c	*	ns	*
CE	1.24 ± 0.27a	0.81 ± 0.14a	0.81 ± 0.35a	0.96 ± 0.32a	1.09 ± 0.27a	1.17 ± 0.17a	ns	ns	ns
NE	0.79 ± 0.17ab	0.51 ± 0.21a	0.46 ± 0.24a	0.64 ± 0.11a	1.16 ± 0.32b	1.07 ± 0.11b	**	ns	*
PE	0.24 ± 0.09ab	0.04 ± 0.01a	0.16 ± 0.11ab	0.14 ± 0.09ab	0.27 ± 0.09b	0.35 ± 0.06b	ns	ns	*

^*a*^ BG: β-glucosidase, CBH: cellobiohydrolase, BX: β-xylosidase, LAP: leucine aminopeptidase, NAGA: N-acetyl-β-D-galactosaminidase, NAG: N-acetyl-β-D-glucosaminidase, AP: alkaline phosphatase, EEA: extracellular enzyme activity, CE: carbon cycling enzyme, NE: nitrogen cycling enzyme, PE: phosphorus cycling enzyme. Significant differences are identified by lowercase letters to the right of the values (one-way analysis of variance; *P* < 0.05). Significance levels of two-way analysis are indicated as follows: ^ns^*P* > 0.05, *P < 0.05, **P < 0.01, ***P < 0.001.

**Fig 6:**
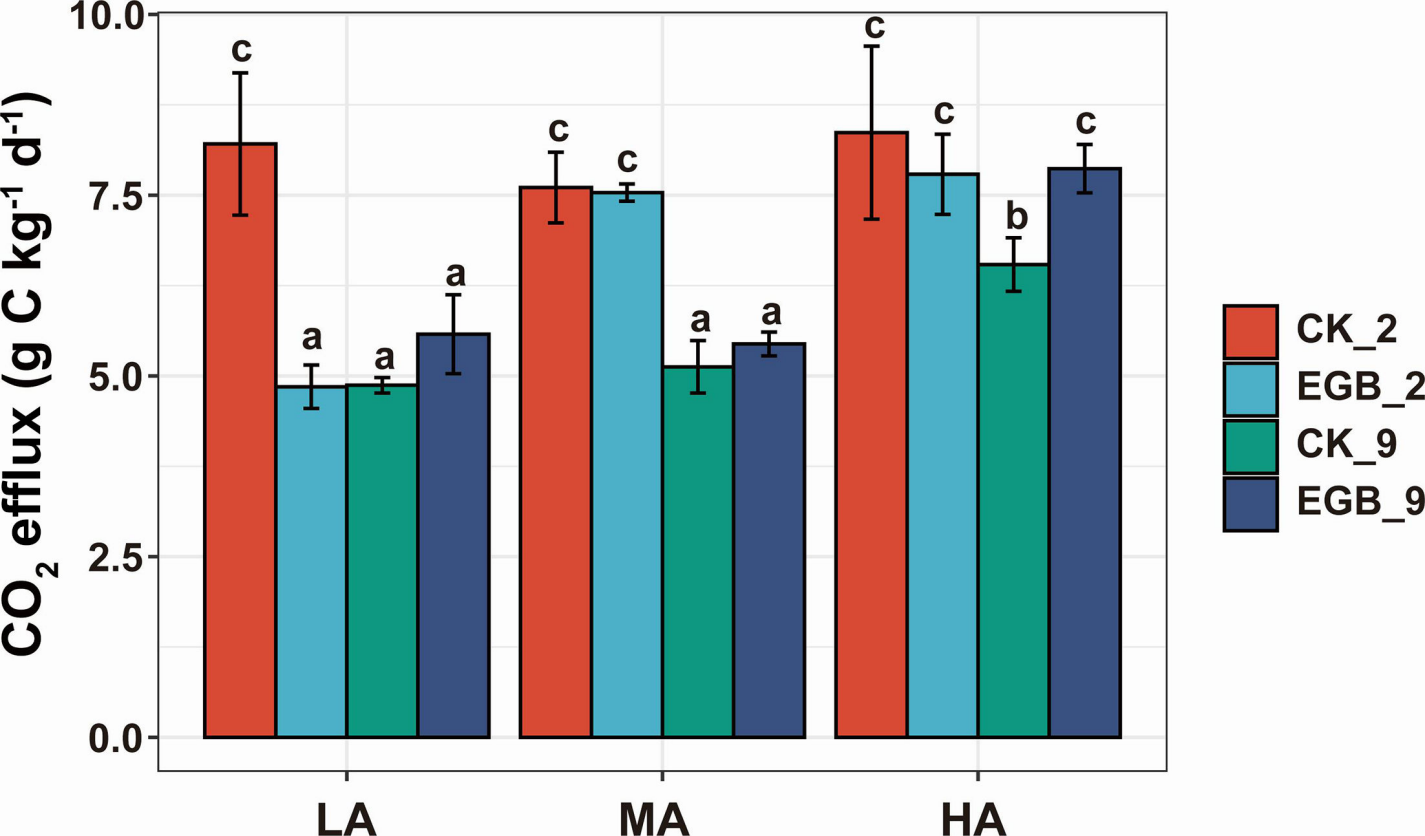
The impact of myxobacterial predation on soil organic carbon mineralization rate (shown as CO_2_ efflux) in soil microcosms across different microbial abundance gradients. CK: control treatment, EGB: myxobacteria treatment; LA: low microbial abundance, MA: medium microbial abundance, HA: high microbial abundance; the number represents incubation time.

Furthermore, myxobacterial predation influenced soil organic carbon mineralization, as measured by CO_2_ efflux (Fig. 6). After 2 days, predation significantly decreased the mineralization rate in all soils, particularly in low microbial abundance soils (*P* < 0.05). After 9 days, however, it increased the mineralization rate across all treatments, with a significant effect in high microbial abundance soils (*P* < 0.05).

### Functional prediction analysis of synthetic bacterial communities in soil microcosms

Functional annotation of synthetic bacterial communities in soil microcosms was conducted using FAPROTAX, based on bacterial absolute abundance data (Fig. S4), which identified 13 functional categories. Significant differences were found in nitrate reduction, fermentation, human-associated processes, and nitrate respiration across various treatments (*P* < 0.05). Myxobacterial predation influenced 10 functional categories, including chemoheterotrophy, fermentation, nitrate respiration, nitrogen respiration, and nitrate reduction, with no effect on aerobic chemoheterotrophy (*P* > 0.05).

Metagenomic sequencing further revealed that myxobacterial predation notably impacted the ecological functions of bacterial communities in soils with high microbial abundance. Among 47 major ecological functions, it suppressed various metabolic processes, particularly lipid metabolism, biosynthesis of secondary metabolites, carbohydrate-binding module synthesis, the central methanogenic pathway, anammox, pyruvate metabolism, and assimilatory sulfate reduction.

**Fig 7:**
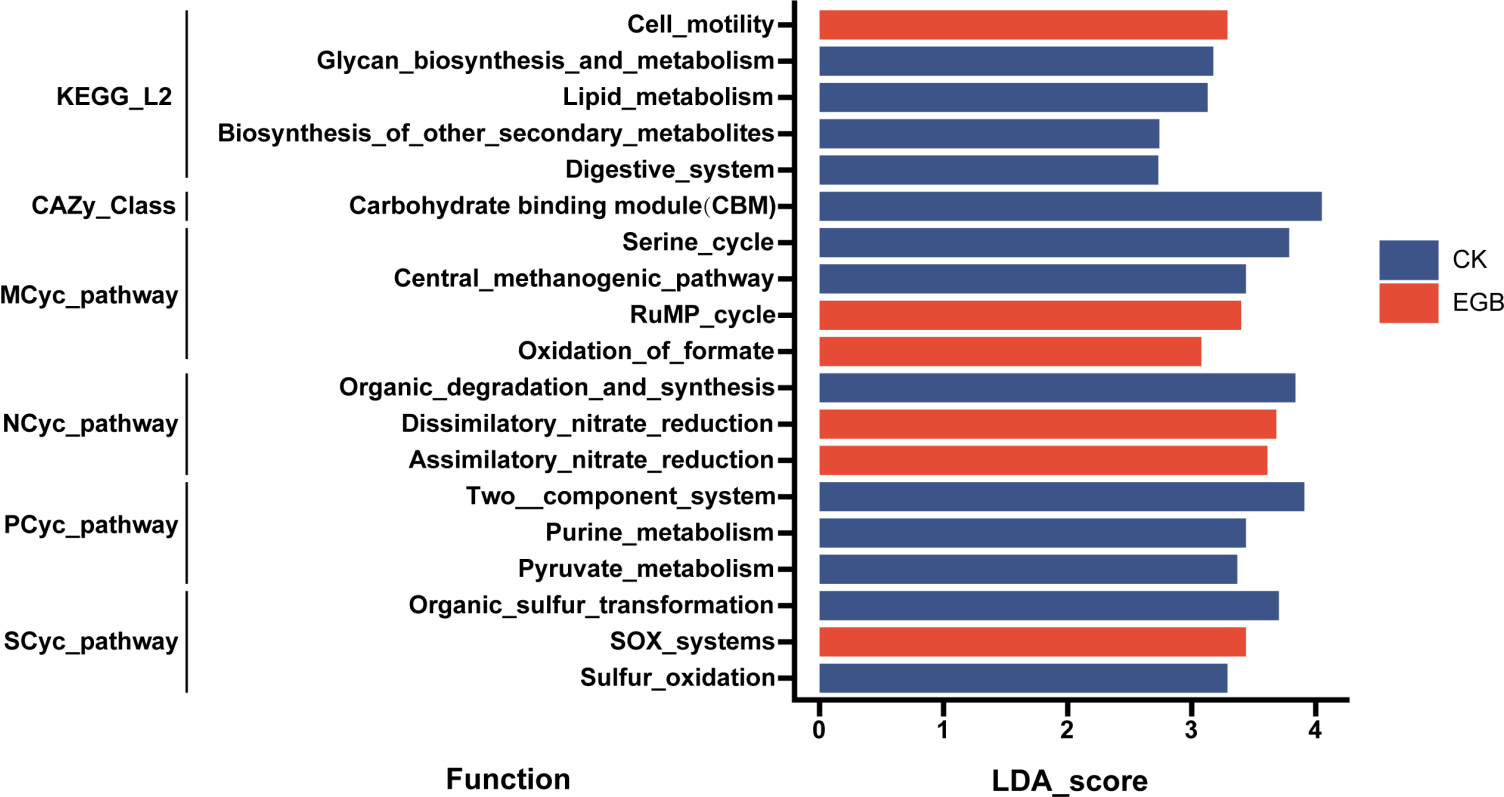
LEfSe analysis of the ecological functional abundance of bacterial communities in the soil microcosm system (linear discriminant analysis [LDA] ≥2.0).

Linear discriminant analysis effect size (LEfSe) analysis (Fig. 7) showed that predation enriched genes for cell motility at the KEGG_L2 level, while controls were enriched in genes for glycan biosynthesis, lipid metabolism, and secondary metabolite biosynthesis. At the CAZy_Class level, carbohydrate-binding module genes were more abundant in control soils (*P* < 0.05). Pathway enrichment analysis indicated that the RuMP cycle and formate oxidation were enhanced under predation, while the serine cycle and methanogenesis were enriched in control groups. Predation also enriched nitrate reduction pathways, whereas organic degradation and sulfur oxidation (SOX system) were more prominent in control soils (*P* < 0.05).

Pathway analysis revealed that the RuMP cycle and formate oxidation were enriched in myxobacteria-treated soils, whereas the serine cycle and central methanogenic pathway were more prevalent in control soils (*P* < 0.05). Myxobacteria treatment also enriched nitrate reduction pathways, while organic degradation pathways were enriched in control soils. At the PCyc_pathway level, sulfur cycling pathways (organic sulfur transformation and sulfur oxidation) were enriched in control soils, with SOX system genes enriched in myxobacteria-treated soils (*P* < 0.05).

## DISCUSSION

Predation constitutes a fundamental ecological process in soil ecosystems, exerting substantial influence on the structure and function of microbial communities (14, 32, 33). In this study, we used a synthetic soil microcosm to investigate the role of *Corallococcus* sp. EGB in shaping microbial community composition and soil ecological functions. Our results provide strain-level evidence that predation regulates bacterial community diversity (H1) and modulates carbon metabolism and nutrient cycling potential (H2), highlighting the ecological significance of predator-mediated regulation in farmland soils.

### Selective regulation of microbial community structure through predation

Predation assays demonstrated differential susceptibility of farmland soil bacteria to *Corallococcus* sp. EGB, with strong predatory activity against *Acinetobacter lwoffii*, *Ensifer sesbaniae*, and *Stenotrophomonas nitritireducens*, and weaker responses against *Microbacterium esteraromaticum*, *Burkholderia vietramienis*, and *Pseudomonas mediterranea*. This variation likely reflects differences in hydrolytic enzyme secretion by the predator, prey metabolic pathways, and physiological states (34). Specifically, myxobacteria may degrade bacterial cell walls through proteases and glycosidases, while prey cell wall composition and growth strategies modulate predation efficiency (35).

In synthetic soil microcosms, *Corallococcus* sp. EGB coexisted with other bacterial strains, and its abundance was generally negatively correlated with most genera. Notably, predation significantly altered the abundance of *Enterobacter*, *Delftia*, and *Stenotrophomonas*, suggesting that selective pressure from *Corallococcus* sp. EGB reshapes community composition. Consequently, this regulation may reduce interspecific competition and enable niche partitioning, ultimately promoting microbial coexistence and maintaining alpha diversity (36). These findings support Hypothesis 1, demonstrating that predation by *Corallococcus* sp. EGB can regulate microbial community structure and diversity under controlled conditions, although extrapolation to other myxobacteria and natural soils requires further validation.

Moreover, susceptibility to predation may depend on bacterial physiological and metabolic states. Rapidly growing and metabolically active bacteria may be more readily encountered and consumed due to higher abundance and metabolic output, while slower-growing bacteria may persist longer but become vulnerable due to reduced defense capabilities rather than being preferentially targeted. Such selective predation reshapes microbial interactions and resource distribution, thereby contributing to community stability and functional diversity. Beyond the scope of this study, other soil predators may exert additional top-down effects, interacting with non-predatory microbes and further shaping community dynamics in natural ecosystems.

### Gradient effect of predation on community diversity and ecological niches

Predation by *Corallococcus* sp. EGB exerts a gradient effect on microbial community diversity, with a limited influence in low-abundance soils but significant reductions in high-abundance soils. Bacterial responses to predation appear to depend on life-history traits: fast-growing, metabolically active r-strategist bacteria (e.g., Proteobacteria) are more readily suppressed, whereas slower-growing k-strategist bacteria (e.g., some nitrogen-fixing taxa) may respond differently, reflecting variable susceptibility rather than absolute resistance.

In resource-rich environments, predation may selectively reduce the abundance of competitive species, disrupting the balance of resource competition, restructuring community dynamics, and affecting community stability and diversity. Such top-down regulation may also indirectly facilitate the coexistence of low-abundance species, highlighting the nuanced role of predation in shaping microbial communities. However, as this study was conducted in a controlled microcosm, the findings may not fully capture the spatial heterogeneity, environmental gradients, and trophic complexity of natural soils. Nevertheless, the observed gradient effects offer valuable insights into the context-dependent roles of *Corallococcus* sp. EGB predation in soil microbial communities.

### Regulatory role and mechanisms of predation on the carbon mineralization rate

Myxobacteria, as key predators in soil microbial food webs, play a crucial role in the carbon cycling within these systems through their interactions with other microbial groups (14). In the synthetic microcosm, microbial metabolism assays revealed a time-dependent effect of predation: short-term inhibition of organic carbon mineralization in low-abundance soils, followed by long-term enhancement after extended incubation. These observations indicate that the influence of predation on carbon mineralization is context-dependent rather than uniformly negative, reflecting interactions between microbial community structure, abundance, and environmental conditions.

Mechanistically, predation may slow the growth of competitive microbial populations, allowing low-abundance taxa with strong carbon metabolic potential to proliferate, thereby accelerating carbon mineralization and enhancing overall soil carbon cycling capacity. By reducing competition among certain microbial populations, predation may improve the metabolic efficiency of remaining groups, contributing to ecosystem-level carbon cycling. No evidence of complete prey resistance to predation was observed during our experiments, likely reflecting the generalist predation strategy of myxobacteria.

Overall, these findings suggest that predation by *Corallococcus* sp. EGB may indirectly modulate soil carbon metabolic potential through community restructuring, consistent with Hypothesis 2. However, given the use of a single predator strain and limited controls, the specific role of predation cannot be fully disentangled from other potential mechanisms. Future studies with additional controls (e.g., non-predatory bacteria, heat-killed predators, or alternative predators) and natural soil environments are required to clarify these ecological effects.

### Broad regulatory effects of predation on soil microbial functions

Myxobacterial communities play a key role in nutrient cycling within farmland soils, particularly in regulating carbon, nitrogen, and phosphorus cycles (9). In the microcosm system, predation by *Corallococcus* sp. EGB primarily reshaped community composition, thereby indirectly influencing the potential functional gene repertoire. As a metagenomic study, observed changes in metabolic pathways reflect shifts in functional potential rather than actual gene expression or metabolic flux.

Predation led to a relative decrease in gene modules associated with lipid metabolism and carbohydrate-binding. This does not imply direct inhibition of these metabolic processes, but rather results from community restructuring, where predation suppresses taxa enriched in these pathways and favors the growth of groups with lower representation of these functions. Consequently, functional attenuation reflects a selective change in gene repertoire rather than suppression of metabolic activity. Notably, although classical studies report that many myxobacteria cannot directly utilize sugars, our predictive analyses indicate that predation indirectly modulates the potential for utilization of phosphorylated sugars and glycosides through shifts in community composition.

In nitrogen cycling, predation shifted the abundance of methanogenic and anammox groups by suppressing dominant taxa and allowing low-abundance, potentially more efficient taxa to expand. These patterns derive from functional prediction and taxonomic profiles rather than direct enzymatic or metabolic measurements. Similar shifts could also occur in predator-free microcosms, emphasizing that both top-down (predation) and bottom-up (microbe-microbe interactions) processes jointly shape the potential functional gene repertoire of soil microbial communities.

Overall, predation by *Corallococcus* sp. EGB indirectly facilitated carbon metabolism and nutrient cycling, likely through restructuring microbial community composition and preserving functional redundancy. Beyond myxobacteria, other bacterial predators—such as *Bdellovibrio*, *Lysobacter*, and *Ensifer*—may exert complementary or synergistic influences through distinct prey spectra and hunting strategies. While our mesocosm approach highlights the ecological implications of predation, future studies incorporating multiple predator groups are needed to disentangle their complementary and potentially synergistic effects on soil microbial functions.

### Conclusion and outlook

This study provides preliminary evidence that a single predatory myxobacterium, *Corallococcus* sp. EGB, can influence microbial community structure and potential carbon and nutrient cycling functions in controlled soil microcosms. Lawn predation assays revealed differential prey susceptibility, indicating functional variability rather than strict selectivity. Predation modulates microbial community structure and diversity and enhances carbon mineralization rates in a time-dependent manner. These findings suggest a potential role of predator-mediated regulation, though they are limited to a single strain and synthetic microcosms. Extrapolation to other myxobacteria or natural soils should therefore be considered tentative and treated as hypotheses for future investigation. Future research should extend beyond strain- and system-specific insights to address the molecular mechanisms of predation (e.g., hydrolytic enzyme secretion, chemical signaling) and evaluate long-term impacts on microbial succession across diverse environmental gradients and spatial scales.

## Data Availability

The raw sequence data generated in this study have been deposited in the NCBI Sequence Read Archive under accession number PRJNA1228988 (https://www.ncbi.nlm.nih.gov/bioproject/PRJNA1228988).

## SUPPLEMENTAL MATERIAL

Graphical abstract aem.01922-25-s0001.pdf Visual depiction of the study.

Supplemental material aem.01922-25-s0002.docx Supplemental methods, Tables S1 to S4, and Fig. S1 to S4.

ASM does not own the copyrights to Supplemental Material that may be linked to, or accessed through, an article. The authors have granted ASM a non-exclusive, world-wide license to publish the Supplemental Material files. Please contact the corresponding author directly for reuse.
