# Supplementary material for: From predation to function: how myxobacteria drive soil microbial community dynamics and ecological functions
Source: Appl Environ Microbiol. 2025 Dec 3;91(12):e01922-25. doi: 10.1128/aem.01922-25 (PMC12724311; doi:10.1128/aem.01922-25)
Supplement: Graphical abstract — Visual depiction of the study. [file aem.01922-25-s0001.pdf]

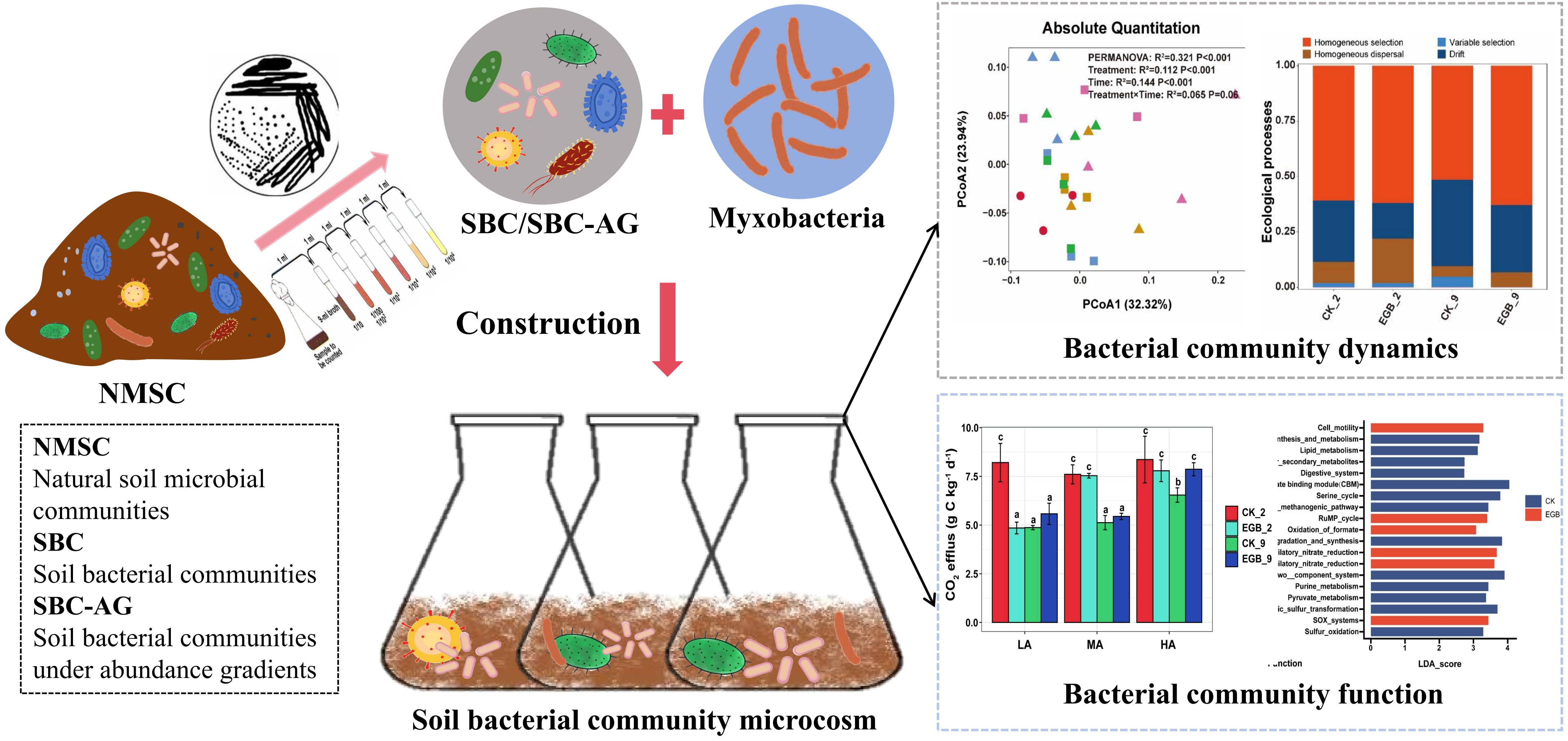
NMSC
NMSC
Natural soil microbial communities
SBC
Soil bacterial communities
SBC-AG
Soil bacterial communities under abundance gradients
SBC/SBC-AG
Myxobacteria
Construction
Soil bacterial community microcosm
Absolute Quantitation
PERMANOVA: R²=0.321 P<0.001
Treatment: R²=0.112 P<0.001
Time: R²=0.144 P<0.001
Treatment×Time: R²=0.065 P=0.06
PCoA1 (32.32%)
PCoA2 (23.94%)
Homogeneous selection
Homogeneous dispersal
Variable selection
Drift
Ecological processes
CK_2
EGB_2
CK_9
EGB_9
Bacterial community dynamics
CO2 efflus (g C kg-1 d-1)
LA
MA
HA
Cell_motility
Lipid_metabolism
Digestive_system
Serine_cycle
RuMP_cycle
Oxidation_of_formate
Purine_metabolism
Pyruvate_metabolism
SOX_systems
Sulfur_oxidation
LDA_score
CK
EGB
Bacterial community function
