# Supplementary material for: From predation to function: how myxobacteria drive soil microbial community dynamics and ecological functions
Source: Appl Environ Microbiol. 2025 Dec 3;91(12):e01922-25. doi: 10.1128/aem.01922-25 (PMC12724311; doi:10.1128/aem.01922-25)
Supplement: Supplemental material — Supplemental methods, Tables S1 to S4, and Fig. S1 to S4. [file aem.01922-25-s0002.docx]

**From Predation to Function: How Myxobacteria Drive Soil Microbial Community Dynamics and Ecological Functions**

**2** **MATERIALS AND METHODS**

**2.3.1 Construction of Synthetic Bacterial Community Soil Microcosm**

To construct the synthetic bacterial community soil microcosm, sterilized Chao soil was first prepared. Ten grams of air-dried, sieved Chao soil were placed into 100 mL serum bottles to prepare 30 soil samples. The soil was subjected to autoclave sterilization at intervals of 0, 12, 24, 48, 72, and 96 hours, with the soil being thoroughly mixed between sterilization periods. After each sterilization cycle, the effectiveness of sterilization was verified by incubating and plating three randomly selected samples, all of which showed no bacterial growth, confirming successful sterilization.

Next, *Corallococcus* sp. EGB and a suspension of 33 types of typical farmland soil bacteria that can stably colonize in sterile Chao soil, were prepared following established methods. The bacteria were cultured in appropriate media, then centrifuged, washed, and resuspended in TPM liquid medium to achieve an optical density OD_600_ of 1.0-1.2. This bacterial suspension was then mixed with sterile soil in 100 mL conical flasks to create a synthetic bacterial community suspension. One milliliter of this suspension was inoculated into the sterilized soil subsamples, adjusting the soil moisture to 60% of the field capacity, and incubated in the dark at 25°C for 3 days to stabilize colonization.

Subsequently, 0.2 mL of either sterile water or *Corallococcus* sp. EGB suspension was added to establish soil microcosms with predatory myxobacteria. Each treatment was performed in triplicate. Destructive sampling was conducted at 0, 2, 5, 9, and 15 days post-inoculation, with soil samples stored at 4 °C for DNA extraction within 24 hours.

**2.3.2 Construction of Soil Microcosms with Different Microbial Abundance Gradients**

To construct soil microcosms with different microbial abundance gradients, 10 grams of air-dried, sieved Chao soil were placed into 100 mL serum bottles to prepare 60 soil subsamples, which were sterilized as described previously. These sterilized soils were then inoculated with bacterial suspensions from non-sterilized Chao soil to form three microbial abundance gradient systems: high abundance (HA, 100 g/g dilution), medium abundance (MA, 10^-3^ g/g dilution), and low abundance (LA, 10^-6^ g/g dilution). Before incubation, the air-dried Chao soil was adjusted to 60% of field capacity with sterile water and incubated in a 30°C constant temperature dark environment for 3 days to restore microbial diversity.

After incubation, 5 grams of the soil were suspended in 100 mL of sterile water and shaken for 1 hour to prepare the soil bacterial suspension. One milliliter of this soil bacterial suspension was inoculated into 10 grams of dry weight sterile Chao soil for each abundance level (HA). The remaining suspension was diluted 10-fold and inoculated into 10 grams of dry weight sterile Chao soil for the medium abundance (MA), and further diluted before inoculation into 10 grams of dry weight sterile Chao soil for the low abundance (LA). All inoculated soils were mixed thoroughly and incubated at 30°C for 48 hours to ensure stable microbial colonization.

Following this, *Corallococcus* sp. EGB was cultured for 48 hours, centrifuged, washed, and resuspended in TPM liquid medium to an OD_600_ of 1.0-1.2. The experiment included a control group (CK) and a treatment group with *Corallococcus* sp. EGB (EGB). Either 0.2 mL of sterile water or *Corallococcus* sp. EGB was added to soils with different abundance gradients to create natural soil microcosms. CO_2_ concentrations were analyzed by sampling the headspace from four subsamples of each abundance gradient at 2 and 9 days post-inoculation. Destructive sampling was also performed. Before each CO_2_ sampling, the headspace was ventilated with air for 10 minutes and resealed. All soil samples were stored at 4°C and DNA extraction was performed within 24 hours of sampling.

**2.4.1 Analysis of Carbon Metabolic Function in Soil Microbial Communities**

To evaluate the diversity of carbon metabolic functions in soil microbial communities, BIOLOG technology was utilized(1). First, 10 grams of fresh soil were added to 100 mL of 0.05 M phosphate buffer and shaken for 30 minutes to thoroughly suspend the soil particles. Then, 1 mL of the suspension was diluted to 10^-3^ concentration. This 10^-3 dilution was transferred to sterilized V-shaped wells, and 150 µL of the diluted solution was added to each well of the BIOLOG ECO plate using an 8-channel pipette. Each sample was tested in triplicate to ensure reproducibility. The ECO plates were incubated at 25°C, and absorbance values were read at wavelengths of 750 nm and 590 nm at the following time points: 24, 48, 72, 96, 120, 144, and 168 hours, using a BIOLOG automatic plate reader. The average well color development (AWCD) was calculated and plotted to track changes over time. This metric was used to assess the carbon metabolic functions of the soil microbial communities.

**2.4.2 Analysis of Extracellular Enzyme Activity Measurement in Soil Microbial Communities**

Extracellular enzyme activity in the soil was measured using a microplate fluorescence assay(2) to reflect the functionality of different extracellular enzymes. This method evaluates enzyme activity by measuring the intensity of fluorescent groups released from fluorescently labeled substrates upon enzymatic cleavage. Specifically, this study measured the activity of seven extracellular enzymes involved in soil carbon, nitrogen, and phosphorus cycles: β-glucosidase (BG), cellobiohydrolase (CBH), and β-xylosidase (BX) primarily involved in the carbon cycle; leucine aminopeptidase (LAP), N-acetyl-β-D-galactosaminidase (NAGA), and N-acetyl-β-D-glucosaminidase (NAG) primarily involved in the nitrogen cycle; and alkaline phosphatase (AP) primarily involved in the phosphorus cycle. First, 100 ml of homogeneous soil suspension was added to a 96-well plate containing 50 ml of substrate and 50 ml of buffer and incubated at 28°C for 30 min. After incubation, fluorescence intensity was measured using a multifunctional microplate reader with an excitation wavelength of 360 nm and an emission wavelength of 450 nm. Each sample was measured in triplicate, alongside standard curves and blank controls (where H_2_O was used instead of soil suspension). Extracellular enzyme activity is typically expressed as the substrate conversion rate per hour per gram of sample, with units of nmol MUF/g/h or nmol AMC/g/h. Total extracellular enzyme activity (EEA) and the activities of enzymes involved in carbon (CE), nitrogen (NE), and phosphorus (PE) cycles were calculated as following formula:

$$Ei=\frac{E}{E_{max}}$$

$$EEA=\sum_{i=1}^{7} Ei$$

$$CE=\sum_{i=1}^{3} Ei$$

$$NE=\sum_{i=3}^{6} Ei$$

$$PE=E_{7}$$

where $E_{i}$ represents the normalized extracellular enzyme activity for a specific enzyme, $E$ is the measured enzyme activity, and $E_{max}$is the maximum enzyme activity among all samples. Specifically, $E1$is for BG, $E2$ for CBH, $E3$ for BX, $E4$ for LAP, $E5$ for NAGA, $E6$ for NAG, and $E7$ for AP.

**2.5.1 16S rRNA Amplicon Sequencing and Data Analysis**

To evaluate the impact of predatory bacteria on bacterial communities in soil microcosms with varying microbial abundance gradients, we employed 16S rRNA amplicon sequencing. DNA was first extracted from soil samples using the FastDNA® Soil Spin Kit (MP Biomedicals, Santa Ana, CA) according to the manufacturer’s instructions. The quality and purity of the extracted DNA were assessed using a NanoDrop 2000 spectrophotometer. Next, the V4-V5 region of the 16S rRNA gene was PCR amplified using a reaction mixture containing FastPfu buffer, dNTPs, primers (515F: 5′-GTGCACAGCMGCCGCGGTAA-3′ and 907R: 5′-CCGTCAATTCCTTTGAGTTT-3′), FastPfu polymerase, and template DNA. The amplification products were verified for quality using 2% agarose gel electrophoresis. The clean PCR products were pooled and sent for sequencing on an Illumina MiSeq PE300 platform. Sequencing data were processed using QIIME 2 (Quantitative Insights Into Microbial Ecology)(3). Chimeric sequences were detected and removed using the Uchime algorithm(4), yielding 6,164,040 high-quality sequences. The sequence depth was normalized to 51,367 reads per sample to ensure uniformity across samples. Operational Taxonomic Units (OTUs) were defined using a 97% similarity threshold. Taxonomic classification was performed using the RDP classifier against the SILVA 132 reference database ([https://www.arb-silva.de/](https://www.arb-silva.de/)), with a minimum confidence threshold of 0.8.

**2.5.2 Absolute Quantitative Sequencing and Data Processing of 16S rRNA Amplicons**

In this study, the 16S rRNA amplicon absolute quantitative sequencing (AQS) technique was employed to analyze the microbial communities within the synthetic bacterial microcosm system(5, 6). Soil samples were first sent to Shanghai Tianhao Biotechnology Co., Ltd. for sequencing. DNA extraction from the samples was performed using the FastDNA® Soil Spin Kit (MP Biomedicals, Santa Ana, CA) according to the manufacturer's instructions. The concentration and integrity of the extracted DNA were assessed using a NanoDrop2000 spectrophotometer (Thermo Fisher Scientific, USA) and agarose gel electrophoresis.

Subsequently, nine insertion sequences with four different concentrations (10^3, 10^4, 10^5, and 10^6 copies/µL) were added to the sample DNA libraries. These insertion sequences included sequences identical to conserved regions of the target 16S rRNA gene and artificially constructed variable regions, which served as internal standards for achieving absolute quantification of the samples. The 16S rRNA gene V4-V5 regions and insertion sequences were amplified and sequenced using the Illumina HiSeq platform.

The preprocessing of sequencing data involved trimming and merging the V4-V5 regions using TrimGalore (http://www.bioinformatics.babraham.ac.uk/projects/trim_galore/) and FLASH2. High-quality sequences were then clustered into operational taxonomic units (OTUs) using the Usearch software platform (http://www.drive5.com/usearch/), with a similarity cutoff of 0.97. OTU classification was further refined using the RDP database. The absolute copy numbers of microbial taxa and functional genes were determined by establishing a standard curve of read counts versus DNA copy numbers. The similarity between bacterial communities was estimated through principal coordinate analysis (PCoA) based on Bray-Curtis dissimilarity. Additionally, the FAPROTAX tool was used to predict the functional profiles of microbial communities in the soil samples.

**2.5.3 Metagenomic DNA Extraction, Sequencing, and Analysis**

For metagenomic sequencing, three replicates were selected from the high microbial abundance soil microcosm samples CK_2 and EGB_2. Soil microbial total DNA was extracted using the OMEGA Mag-Bind Soil DNA Kit (OMEGA Bio-Tek, Norcross, GA, USA) according to the manufacturer's instructions and stored at -20°C. The concentration and quality of the extracted DNA were assessed using a Qubit™ 4 fluorometer and agarose gel electrophoresis. Subsequently, metagenomic libraries with an insert size of 400 bp were prepared using the Illumina TruSeq Nano DNA LT Library Preparation Kit and sequenced on the Illumina NovaSeq platform (Illumina, USA) using a PE150 strategy.

In the metagenomic data analysis, the raw sequencing data were first subjected to quality filtering. Adapter sequences were removed using Cutadapt (v1.2.1)(7), and low-quality sequences were trimmed using the sliding window algorithm in fastp. The quality-filtered sequences were then classified using Kraken2(8), based on a RefSeq-derived database. Alleles longer than 300 bp were clustered using the "easy-lin-clust" mode in mmseqs2, with a sequence identity threshold of 0.95 and a coverage threshold of 90% for shorter alleles. Further, the "taxonomy" mode in mmseqs2(9) was used to compare non-redundant alleles against the NCBI-nt database to determine their lowest common ancestor taxonomy and to exclude viral alleles.

Genes within the alleles were predicted using MetaGeneMark(10). High-quality reads were mapped to the predicted gene sequences using salmon in the "-meta- minScoreFraction=0.55" mode, and data were normalized using counts per million (CPM). Functional annotation of the predicted gene sequences was performed by aligning amino acid sequences with the Kyoto Encyclopedia of Genes and Genomes (KEGG) database, with a focus on genes related to carbon (C), nitrogen (N), phosphorus (P), and sulfur (S) cycles. Additionally, the sequences were compared to the Carbohydrate-Active Enzymes (CAZy) database, which includes glycoside hydrolases (GH), glycosyltransferases (GT), polysaccharide lyases (PL), carbohydrate esterases (CE), auxiliary activities (AA), and carbohydrate-binding modules (CBMs). Furthermore, genes related to methane, nitrogen, phosphorus, and sulfur cycles were analyzed using the MCyc, NCyc, PCyc, and SCyc databases, respectively.

1. Sofo A, Ricciuti P. 2019. A Standardized Method for Estimating the Functional Diversity of Soil Bacterial Community by Biolog® EcoPlatesTM Assay-The Case Study of a Sustainable Olive Orchard. Applied Sciences-Basel 9.

2. Marx M-C, Wood M, Jarvis SC. 2001. A microplate fluorimetric assay for the study of enzyme diversity in soils. Soil Biology and Biochemistry 33:1633-1640.

3. Caporaso JG, Kuczynski J, Stombaugh J, Bittinger K, Bushman FD, Costello EK, Fierer N, Pena AG, Goodrich JK, Gordon JI, Huttley GA, Kelley ST, Knights D, Koenig JE, Ley RE, Lozupone CA, McDonald D, Muegge BD, Pirrung M, Reeder J, Sevinsky JR, Tumbaugh PJ, Walters WA, Widmann J, Yatsunenko T, Zaneveld J, Knight R. 2010. QIIME allows analysis of high-throughput community sequencing data. Nature Methods 7:335-336.

4. Edgar RC, Haas BJ, Clemente JC, Quince C, Knight R. 2011. UCHIME improves sensitivity and speed of chimera detection. Bioinformatics 27:2194-2200.

5. Smets W, Leff JW, Bradford MA, McCulley RL, Lebeer S, Fierer N. 2016. A method for simultaneous measurement of soil bacterial abundances and community composition via 16S rRNA gene sequencing. Soil Biology & Biochemistry 96:145-151.

6. Tkacz A, Hortala M, Poole PS. 2018. Absolute quantitation of microbiota abundance in environmental samples. Microbiome 6.

7. Martin M. 2011. Cutadapt removes adapter sequences from high-throughput sequencing reads. EMBnetjournal 17:10-12.

8. Wood DE, Lu J, Langmead B. 2019. Improved metagenomic analysis with Kraken 2. Genome Biology 20.

9. Steinegger M, Söding J. 2017. MMseqs2 enables sensitive protein sequence searching for the analysis of massive data sets. Nat Biotechnol.

10. Wenhan Z, Alexandre L, Mark B. 2010. Ab initio gene identification in metagenomic sequences. Nucleic Acids Research 38:e132.

Table S1. Information on 33 potential prey bacteria

| Potential prey bacteria | Homology (%) | Taxonomy (Phylum) | Gram characteristics | OD_600_ (A) |
| --- | --- | --- | --- | --- |
| *Microbacterium azadirachtae* | 99.93 | Actinobacteria | Positive | 1.16 |
| *Microbacterium hydrocarbonoxydans* | 99.93 | Actinobacteria | Positive | 1.37 |
| *Microbacterium esteraromaticum* | 99.38 | Actinobacteria | Positive | 1.38 |
| *Rhizobium daejeonense* | 99.95 | Actinobacteria | Negative | 1.23 |
| *Acinetobacter lwoffii* | 99.67 | Bacteroidetes | Negative | 1.66 |
| *Chitinoph·agaoryzae* | 99.52 | Bacteroidetes | Negative | 1.23 |
| *Chryseobacterium bernardetii* | 98.91 | Bacteroidetes | Negative | 1.36 |
| *Chryseobacterium daecheongense* | 98.69 | Bacteroidetes | Negative | 1.35 |
| *Chryseobacterium cucumeris* | 99.59 | Bacteroidetes | Positive | 1.38 |
| *Flavobacterium naphthae* | 99.38 | Bacteroidetes | Negative | 1.33 |
| *Olivibacter jilunii* | 99.39 | Bacteroidetes | Negative | 1.19 |
| *Sphingobacterium siyangense* | 98.71 | Bacteroidetes | Negative | 1.36 |
| *Sphingobacterium sp. GF2B* | 99.73 | Bacteroidetes | Negative | 1.38 |
| *Bacillus altitudinis* | 99.86 | Firmicutes | Positive | 1.35 |
| *Bacillus cereus* | 99.95 | Firmicutes | Positive | 1.32 |
| *Bacillus subtilis* | 99.93 | Firmicutes | Positive | 1.32 |
| *Bacillus velezensis* | 99.71 | Firmicutes | Positive | 1.32 |
| *Lysinibacillus sphaericus* | 99.93 | Firmicutes | Negative | 1.36 |
| *Lysinibacillus xylanilyticus* | 99.29 | Firmicutes | Positive | 1.4 |
| *Achromobacter xylosoxidans* | 99.8 | Proteobacteria | Negative | 1.31 |
| *Burkholderia vietramienis* | 99.62 | Proteobacteria | Negative | 1.61 |
| *Delftia tsuruhatensis* | 99.11 | Proteobacteria | Negative | 1.31 |
| *Diaphorobacter ruginosibacter* | 99.86 | Proteobacteria | Negative | 1.42 |
| *Enterobacter cloacae* | 99.73 | Proteobacteria | Negative | 1.37 |
| *Enterobacter hormaechei* | 99.8 | Proteobacteria | Negative | 1.36 |
| *Pseudomonas mediterranea* | 99.93 | Proteobacteria | Negative | 1.35 |
| *Pseudomonas oryzihabitans* | 99.8 | Proteobacteria | Negative | 1.65 |
| *Pseudomonas plecoglossicida* | 99.72 | Proteobacteria | Negative | 1.38 |
| *Pseudoxanthomonas japonensis* | 99.41 | Proteobacteria | Negative | 1.16 |
| *Ralstonia sp.* | 99.3 | Proteobacteria | Negative | 1.34 |
| *Stenotrophomonas geniculata* | 99.59 | Proteobacteria | Positive | 1.37 |
| *Stenotrophomonas maltophilia* | 99.8 | Proteobacteria | Negative | 1.35 |
| *Stenotrophomonas nitritireducens* | 99.66 | Proteobacteria | Negative | 1.35 |

Table S2. Distribution of differential bacterial groups in synthetic bacterial microcosms

| **Absolute abundance** | ***Enterobacter*** | ***Delftia*** | ***Stenotrophomonas*** | ***Burkholderia*** | ***Lysinibacillus*** | ***Chryseobacterium*** |
| --- | --- | --- | --- | --- | --- | --- |
| PS | 750002827±34280562.19b | 4329866.67±1625032.25a | 47351413.67±7540972.35a | 1038974.67±193343.29a | 69219.33±37735.94ab | 142347376.67±21756309.05ab |
| CK_2 | 623342428.33±22413938.56a | 4733587.33±555157.1ab | 49579957.67±1433277.87a | 1149117.33±201999.09a | 73602±7051.09ab | 168423591.33±17981571.69abcd |
| CK_5 | 1010958617.67±22413938.56c | 5985091.33±555157.1ab | 55160442±1433277.87a | 2000757.33±201999.09c | 117810.33±7051.09bc | 229228073.33±17981571.69bcd |
| CK_9 | 985369239.67±14687639.07c | 5400746±703831.54ab | 54872951.33±4836590.89a | 1667999±254932.78ab | 100527.67±12664.07b | 162429404.33±50016363.07abc |
| CK_15 | 1104531397.67±112149009.48c | 11392477±3653291.78cd | 83917656.67±18286518.62cd | 2663130.67±477654.59d | 113542±22727.9bc | 130667015±26026077.61a |
| EGB_2 | 1000866139±91671834.06c | 9179670.67±1053949.18bc | 77097500.33±3235736.76bc | 1619061±210102.54ab | 125566.67±37197.23bc | 243495078.33±96812574.8cd |
| EGB_5 | 510204891.67±24792018.88a | 5415172±887794.07ab | 55839902±12397277.43a | 1402556±367658.52ab | 24032.67±20998.67a | 94999889.33±25072082.37a |
| EGB_9 | 746638008.67±50119419.59b | 6398042.33±771006.03ab | 60809613.67±3946654.7ab | 1467958.67±274441.37ab | 66762.67±11593.07ab | 115102025.67±3200353.96a |
| EGB_15 | 829610637.67±72591890.32b | 15437682.67±5617798.64d | 100922710.67±24659801.94d | 1947972±551388.09c | 164572.33±68108.92c | 257076731.67±42306537.99d |
| Myxobacteria Treatment(M) | *** | * | ** | ns | ns | ns |
| Inoculation time(T) | *** | *** | ** | *** | * | ns |
| M*T | *** | ns | ns | ** | * | *** |
| **Absolute abundance** | ***Bacillus*** | ***Diaphorobacter*** | ***Flavobacterium*** | ***Pseudoxanthomonas*** | ***Olivibacter*** |  |
| PS | 440745.33±172229.1a | 14804257.33±8791393a | 5051990±1464220.75a | 161237417.33±25780549a | 32002510.67±3246280.65bc |  |
| CK_2 | 651577.33±393869.06ab | 14210280.33±1425590.36a | 7019877±3590168.44a | 198314131.67±18772397.69ab | 29382129.33±2550709.46abc | |
| CK_5 | 1370441.67±393869.06bc | 21057516.67±1425590.36ab | 11889592.33±3590168.44a | 212894052±18772397.69ab | 30031309.67±2550709.46abc | |
| CK_9 | 1051550.67±134813.12ab | 18124836.33±300958.81ab | 9103674±2881498.28a | 219503277.67±43657051.7ab | 22395856±8751865.06ab |  |
| CK_15 | 1276582.67±287200.69bc | 40643826.33±15033583.87bc | 33856258.33±19946842.41bc | 355166871.67±20636891.3c | 19683993±1326122.74a |  |
| EGB_2 | 1233715.33±444879.64bc | 31135861.67±7871760.43ab | 17680148.33±7740281.96ab | 272767876.67±70144100.14bc | 35930433.33±12098987.93c |  |
| EGB_5 | 372567±190294.48a | 16193164±1222554.07a | 5744094±3397288.6a | 266050981.33±83396102.67bc | 22385210.67±4259757.12ab |  |
| EGB_9 | 650982.67±70277.59ab | 21456425±4846215.8ab | 13210907.67±3377286.23a | 255218139±40037931.63ab | 18553588.33±1131854.42a |  |
| EGB_15 | 1877317±1015391.52d | 54733249±31098423.6c | 37731868±23856380.62c | 261955500±51881858.4bc | 30529998±8411168.71abc |  |
| Myxobacteria Treatment(M) | ns | ns | ns | ns | ns |  |
| Inoculation time(T) | * | * | * | * | * |  |
| M*T | ** | ns | ns | ns | ns |  |
|  |  |  |  |  |  |  |
|  |  |  |  |  |  |  |
| **Relative abundance** | ***Olivibacter*** | ***Delftia*** | ***Enterobacter*** | ***Pseudoxanthomonas*** | ***Stenotrophomonas*** | ***Chitinophaga*** |
| PS | 8544.67±679.31d | 1435±477.53a | 50232.33±2478.17bcd | 14331.33±1981.06a | 11333.67±1524.79ab | 18502±2118.68bc |
| CK_2 | 7936.33±611.68cd | 1574±242.51a | 42962.67±1786.24ab | 17998.67±1005.38abc | 11925.67±543.38abc | 18115.33±2918.63bc |
| CK_5 | 6283±690.95bc | 1563.33±56.89a | 53405.67±5557.75cd | 14997.33±4666.53ab | 10391.67±752.34a | 15054±2967.62ab |
| CK_9 | 5197.33±1831.83ab | 1594.67±173.87a | 57611.33±6674.21d | 16888.67±2258.08abc | 11527.33±419.49ab | 14492±3392.55ab |
| CK_15 | 3534.33±178.4a | 2549.33±783.79bc | 50102.33±7064.02bcd | 21367±2125.89bcd | 13530.67±2655.47bcd | 14617.67±2907.82ab |
| EGB_2 | 6541.33±1596.92bcd | 2112.67±302.99ab | 46300±4728.01abc | 16754.67±4799.91abc | 12693±1326.12abc | 14232.67±1786.42ab |
| EGB_5 | 6569.67±472.88bcd | 1992.67±277.07ab | 38912.67±6931.14a | 25873.33±4347.68d | 14604.67±1323.3cd | 20863±1954.03c |
| EGB_9 | 4716.67±493.64ab | 2027.33±263.18ab | 47576.67±1299.81abc | 21521±2389.8cd | 13884±1564.99bcd | 17212±1978.46bc |
| EGB_15 | 5428.33±1800.97ab | 3301±878.66c | 37919.33±6367.96a | 15778±4192.29abc | 15714.67±2290.02d | 12161.33±2058.96a |
| Myxobacteria Treatment(M) | ns | * | ** | ns | *** | ns |
| Inoculation time(T) | *** | * | ns | ns | ns | ns |
| M*T | ns | ns | * | ** | ns | ** |
| **Relative abundance** | ***Lysinibacillus*** | ***Burkholderia*** | ***Chryseobacterium*** | ***Microbacterium*** | ***Bacillus*** | ***Flavobacterium*** |
| PS | 47.67±24.7b | 327±69.31a | 16086.67±2464.57ab | 657±131.05a | 312.33±126.62ab | 1653.67±454.22a |
| CK_2 | 50.33±9.07b | 350.67±56.01ab | 18010.67±1399.98abc | 363.67±141.51ab | 445.67±277.89abcd | 2293.33±1216.86a |
| CK_5 | 68.33±15.14b | 497.67±111.13bc | 18711±4356.2abc | 348.67±74.66ab | 743.67±32.13cd | 3013.33±817a |
| CK_9 | 65±9b | 463.67±73.89abc | 14506.67±4226.01ab | 381.67±117.51ab | 654±102.62bcd | 2616.67±921.35a |
| CK_15 | 57±15.62b | 564.67±106.21c | 10705.67±3989.33a | 391.33±133.57ab | 595.67±130.22abcd | 7346.33±4153.45c |
| EGB_2 | 58.67±4.73b | 350.33±40.08ab | 20067±8599.71bc | 322.33±74.88a | 567.67±110.39abcd | 4065.67±2057.46ab |
| EGB_5 | 19±17.06a | 468±81.85abc | 11997.67±2951.16ab | 560±87.62bc | 284.67±149.5a | 2081.67±1215.56a |
| EGB_9 | 43.67±6.66ab | 440±68.51abc | 12796.33±2049.85ab | 567±125.05bc | 432±16.37abc | 4133±1202.69ab |
| EGB_15 | 71±24.25b | 382.67±79.22ab | 25512.67±6629.42c | 360±166.5ab | 793.67±386.17d | 7829±4564.68c |
| Myxobacteria Treatment(M) | ns | ns | ns | ns | ns | ns |
| Inoculation time(T) | ns | ** | ns | ** | ns | * |
| M*T | ** | ns | ** | ns | * | ns |

Note: lowercase letters represent significant differences,* represents P<0.05, ** represents P<0.01,*** represents P<0.001; M: Myxobacteria Treatment, T: Inoculation time, M*T: Myxobacteria Treatment*Inoculation time; Significant differences are identified by lowercase letters to the right of the values (one-way analysis of variance; P <0.05)， Significance levels of two-way analysis are indicated as * P < 0.05, **P < 0.01, ***P < 0.001.

Table S3. Loadings for principal coordinate analysis of carbon source utilization by bacterial communities of different treatments in soil microcosms.

| **Carbon Source** | **PCoA1** | **PCoA2** |
| --- | --- | --- |
| **Carbohydrates** |  |  |
| D-Fibroin | -0.645** | 0.673** |
| β-Methyl-D-Glucoside | -0.244 | 0.747*** |
| i-Erythritol | 0.681** | 0.472* |
| D-Mannitol | -0.298 | 0.639** |
| N-Acetyl-D-Glucosamine | -0.712*** | 0.437 |
| Glucose-1-phosphate | 0.938*** | 0.228 |
| D, L-α-Glycerophosphate | 0.26 | 0.712*** |
| D-Galacturonic Acid γ-Lactone | 0.513 | 0.316 |
| **Amino Acids** |  |  |
| L-Asparagine | -0.428 | -0.592** |
| L-Threonine | 0.922*** | -0.067 |
| Glycyl-L-Glutamic Acid | -0.172 | 0.325 |
| **Carboxylic Acids** |  |  |
| D-Galacturonic Acid | -0.007 | 0.522* |
| γ-Hydroxybutyric Acid | -0.775*** | 0.001 |
| Itaconic Acid | -0.721*** | 0.596** |
| α-Ketobutyric Acid | 0.875*** | 0.092 |
| D-Malic Acid | 0.168 | 0.688** |
| **Amines** |  |  |
| Phenethylamine | 0.715*** | -0.272 |
| Putrescine | 0.428 | 0.818*** |
| **Phenolic Acids** |  |  |
| 4-Hydroxybenzoic acid | 0.849*** | 0.358 |
| **Polymers** |  |  |
| Tween 40 | 0.69** | -0.301 |
| Tween 80 | 0.626** | -0.054 |
| α-cyclodextrin | 0.143 | 0.696** |
| Glycogen | -0.201 | -0.63** |

Note: The number represents the correlation coefficient, * represents P<0.05, ** represents P<0.01,*** represents P<0.001.

Table S4. Analysis of Soil Metabolic Functions Based on Different Functional Levels

| Function | CK | EGB | P value (t test) | EGB vs CK |
| --- | --- | --- | --- | --- |
| KEGG_L2 |  |  |  |  |
| Lipid metabolism | 0.0481±0.0003 | 0.0469±0.0006 | **0.025** | Down |
| Biosynthesis of other secondary metabolites | 0.0485±0.0007 | 0.047±0.0007 | **0.006** | Down |
| Metabolism of terpenoids and polyketides | 0.0491±0.0003 | 0.0472±0.0028 | 0.408 | Down |
| Glycan biosynthesis and metabolism | 0.052±0.0004 | 0.0488±0.0017 | 0.066 | Down |
| Metabolism of other amino acids | 0.0535±0.0003 | 0.0538±0.0002 | 0.348 | Up |
| Energy metabolism | 0.0553±0.0009 | 0.055±0.0001 | 0.679 | Down |
| Xenobiotics biodegradation and metabolism | 0.0613±0.0055 | 0.0613±0.0013 | 0.988 | Down |
| Metabolism of cofactors and vitamins | 0.1004±0.0014 | 0.0992±0.0006 | 0.139 | Down |
| Carbohydrate metabolism | 0.1349±0.0013 | 0.1337±0.0019 | 0.41 | Down |
| Amino acid metabolism | 0.1627±0.0019 | 0.1628±0.0004 | 0.924 | Up |
| CAZy_Class |  |  |  |  |
| Auxiliary Activities (AA) | 0.0179±0.0004 | 0.0186±0.002 | 0.134 | Up |
| Polysaccharide lyase (PL) | 0.02±0.0005 | 0.0196±0.0003 | 0.2 | Down |
| Carbohydrate esterase (CE) | 0.0527±0.0002 | 0.0528±0.0002 | 0.519 | Up |
| Carbohydrate Binding Module (CBM) | 0.1608±0.0025 | 0.1529±0.0043 | **0.046** | Down |
| Glycoside hydrolase (GH) | 0.3723±0.0013 | 0.3742±0.0024 | 0.3 | Up |
| Glycosyltransferase (GT) | 0.3763±0.0026 | 0.3819±0.0051 | 0.144 | Up |
| MCyc_pathway |  |  |  |  |
| Methylotrophic methanogenesis | 0.0222±0.0015 | 0.0206±0.0001 | 0.21 | Down |
| Hydrogenotrophic methanogenesis | 0.0236±0.001 | 0.0247±0.0008 | 0.403 | Up |
| Oxidation of formate | 0.031±0.0004 | 0.0322±0.0004 | 0.143 | Up |
| Oxidation of formaldehyde | 0.0487±0.0003 | 0.0478±0.0007 | 0.296 | Down |
| Oxidation of merthane and C1 compounds | 0.0781±0.0034 | 0.077±0.0018 | 0.723 | Down |
| RuMP cycle | 0.0777±0.0004 | 0.08±0.0012 | 0.14 | Up |
| Central methanogenic pathway | 0.0812±0.0005 | 0.0792±0.001 | **0.045** | Down |
| Anaerobic oxidation of methane (AOM) | 0.1444±0.0007 | 0.1442±0.0017 | 0.865 | Down |
| Aceticlastic methanogenesis | 0.1668±0.0051 | 0.1723±0.0026 | 0.096 | Up |
| Serine cycle | 0.3262±0.0012 | 0.3219±0.0023 | 0.069 | Down |
| NCyc_pathway |  |  |  |  |
| Nitrification | 0.0004±0.0001 | 0.0001±0.0001 | 0.102 | Down |
| Anammox | 0.0004±0.0001 | 0.0002±0.0001 | **0.039** | Down |
| Nitrogen fixation | 0.0018±0.0001 | 0.0016±0.0001 | 0.301 | Down |
| Assimilatory nitrate reduction | 0.1001±0.0016 | 0.1047±0.0027 | 0.201 | Up |
| Dissimilatory nitrate reduction | 0.1014±0.0031 | 0.1094±0.0017 | 0.102 | Up |
| Denitrification | 0.1309±0.0024 | 0.1281±0.0027 | 0.197 | Down |
| Organic degradation and synthesis | 0.6643±0.0025 | 0.6549±0.0059 | 0.164 | Down |
| PCyc_pathway |  |  |  |  |
| Phosphotransferase system | 0.0165±0.0003 | 0.0171±0.0003 | 0.261 | Up |
| Oxidative phosphorylation | 0.0257±0.0003 | 0.0277±0.0019 | 0.166 | Up |
| Phosphonate and phosphinate metabolism | 0.0397±0.0004 | 0.0395±0.0011 | 0.775 | Down |
| Organic phosphoester hydrolysis | 0.0428±0.0002 | 0.0416±0.0015 | 0.321 | Down |
| Pyruvate metabolism | 0.0473±0.0007 | 0.0458±0.0007 | **0.004** | Down |
| Pentose phosphate pathway | 0.088±0.0015 | 0.0877±0.0011 | 0.824 | Down |
| Transporters | 0.0952±0.0035 | 0.1001±0.0046 | 0.253 | Up |
| Pyrimidine metabolism | 0.1491±0.0012 | 0.1482±0.0027 | 0.713 | Down |
| Purine metabolism | 0.2121±0.001 | 0.2084±0.0026 | 0.091 | Down |
| Two-component system | 0.2376±0.0059 | 0.2297±0.0008 | 0.116 | Down |
| SCyc_pathway |  |  |  |  |
| Sulfur oxidation | 0.0385±0.0005 | 0.0376±0.0001 | 0.079 | Down |
| Assimilatory sulfate reduction | 0.1762±0.001 | 0.1735±0.0014 | **0.03** | Down |
| Link between inorganic and organic sulfur transformation | 0.2023±0.0006 | 0.2022±0.0014 | 0.962 | Down |
| Organic sulfur transformation | 0.307±0.0009 | 0.3045±0.0008 | 0.137 | Down |


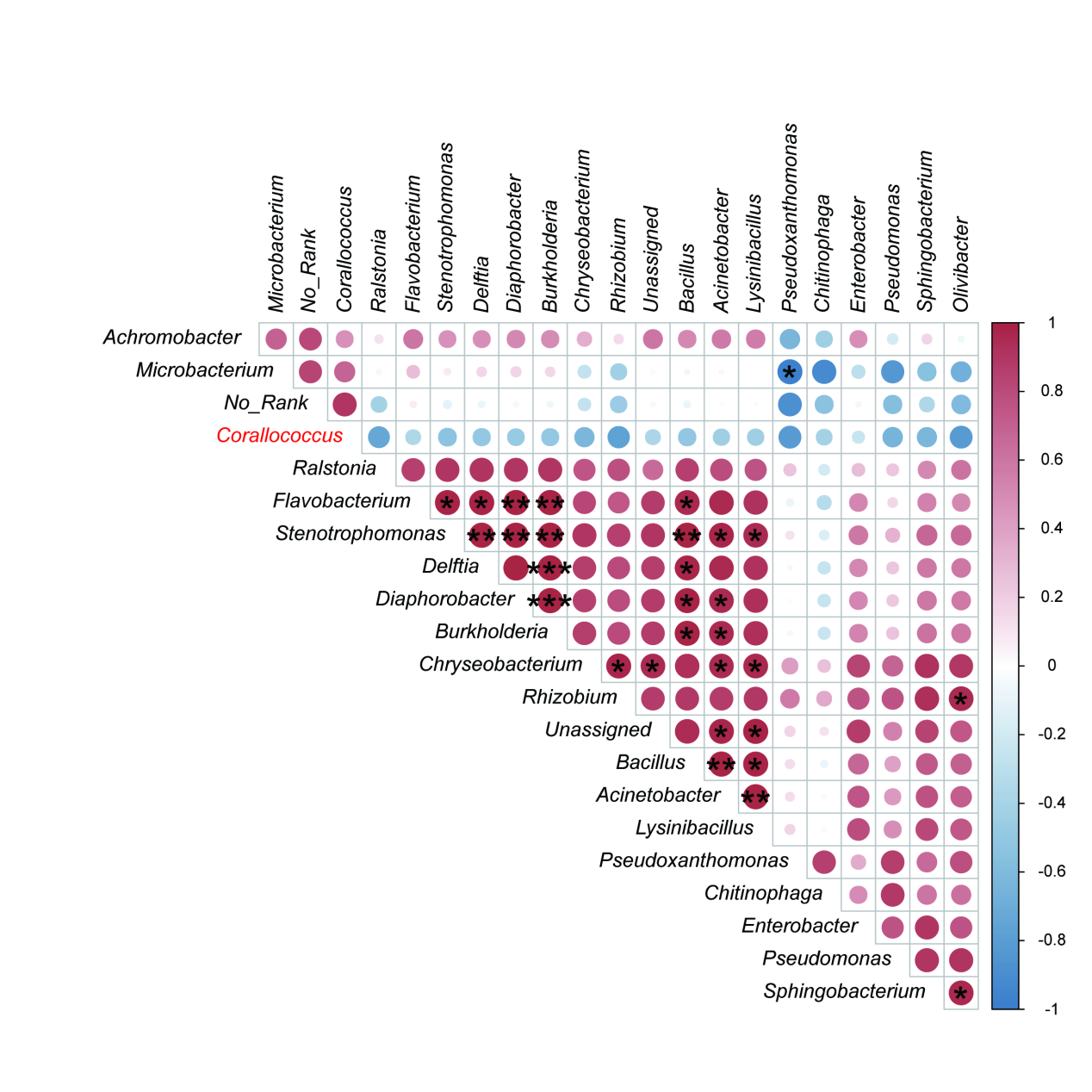


Figure S1. Correlation analysis of different bacterial genera in synthetic bacterial community soil microcosm systems


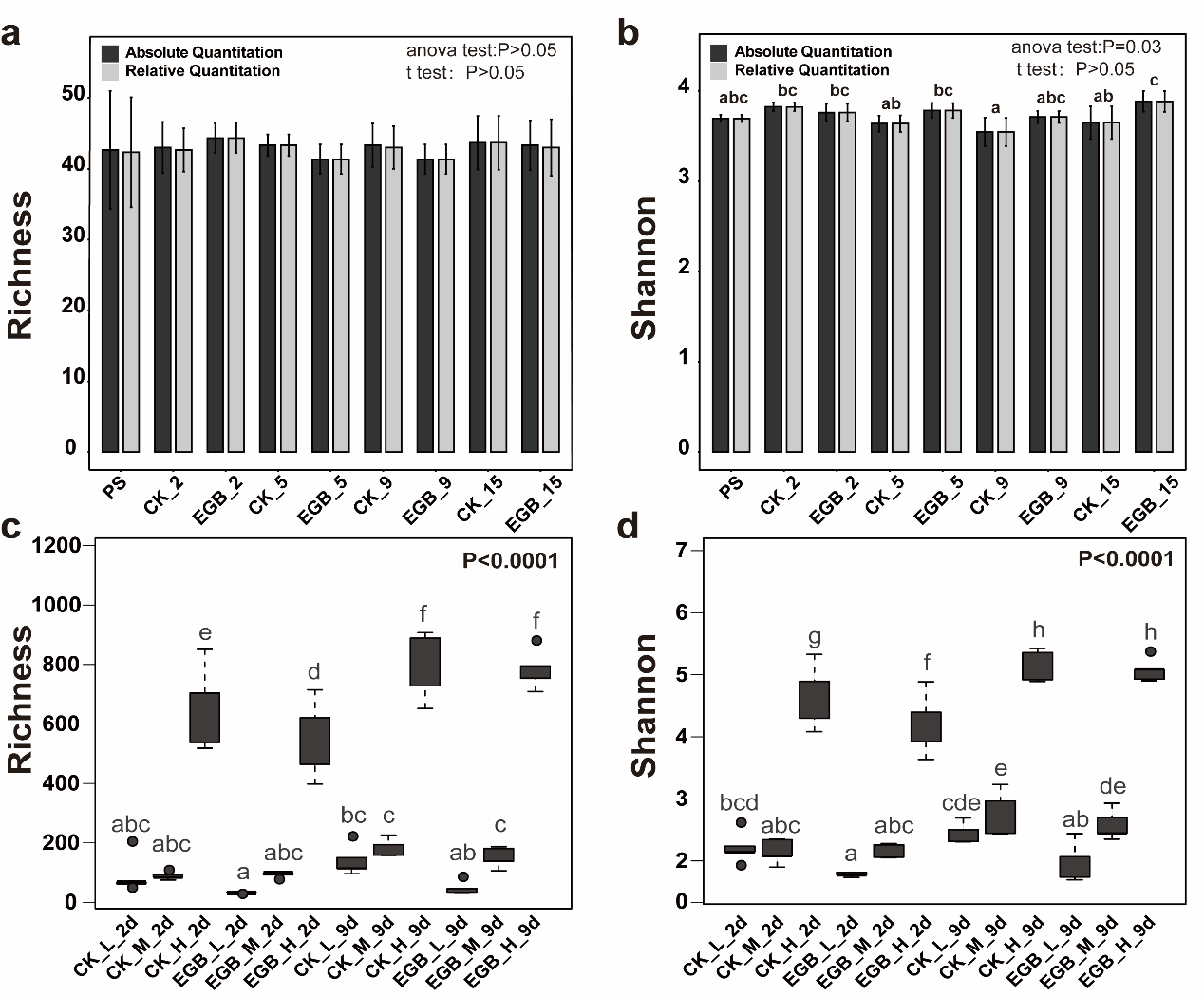


Figure S2. Bacterial community diversity in soil microcosm systems. richness (a) and diversity (b) of bacterial communities in synthetic bacterial community soil microcosms. Richness (c) and diversity (d) of bacterial communities in soil microcosms with different microbial abundance gradients. CK: control treatment, EGB: myxobacteria treatment; L: low microbial abundance, M: medium microbial abundance, H: high microbial abundance; The number represents incubation time.


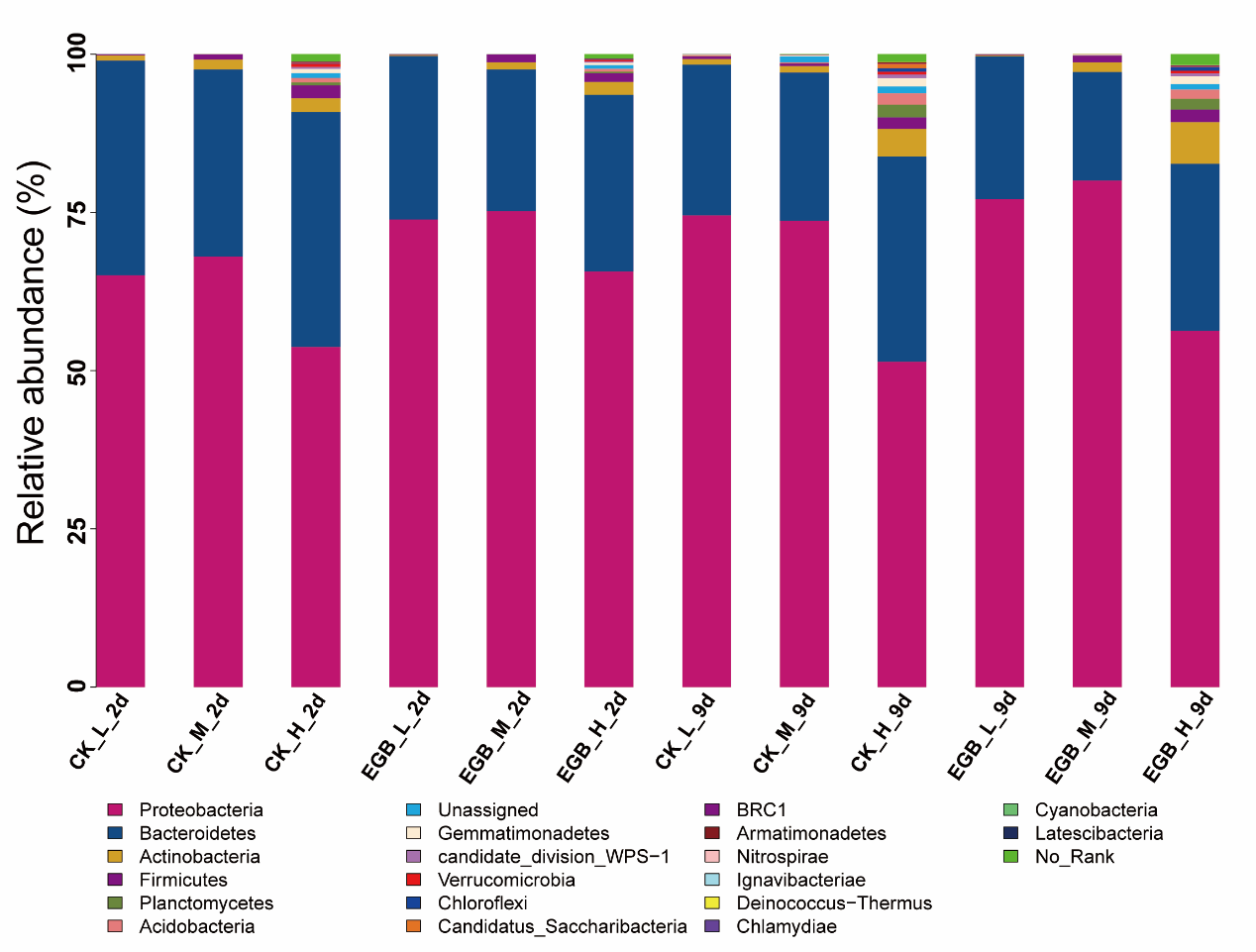


Figure S3. Bacterial community composition in soil microcosms with different microbial abundance gradients at the phylum level. CK: control treatment, EGB: myxobacteria treatment; L: low microbial abundance, M: medium microbial abundance, H: high microbial abundance; The number represents incubation time.


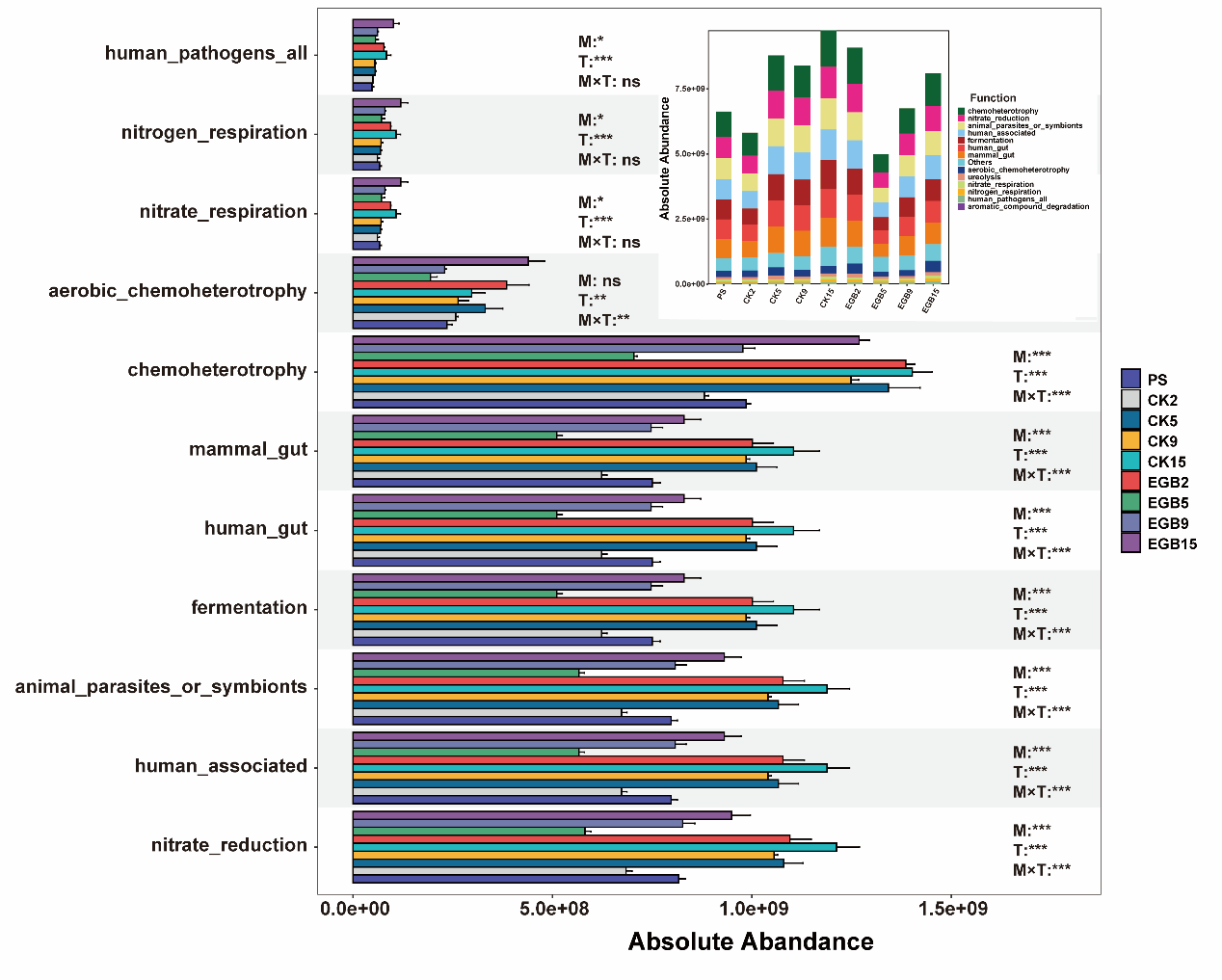


Figure S4. Differences in predicted functional groups among different soil treatments in synthetic bacterial community microcosm systems. M: Myxobacteria Treatment, T: Inoculation time, M*T: Myxobacteria Treatment*Inoculation time.
